# Preferential uptake of antioxidant carbon nanoparticles by T lymphocytes for immunomodulation

**DOI:** 10.1038/srep33808

**Published:** 2016-09-22

**Authors:** Redwan Huq, Errol L. G. Samuel, William K. A. Sikkema, Lizanne G. Nilewski, Thomas Lee, Mark R. Tanner, Fatima S. Khan, Paul C. Porter, Rajeev B. Tajhya, Rutvik S. Patel, Taeko Inoue, Robia G. Pautler, David B. Corry, James M. Tour, Christine Beeton

**Affiliations:** 1 Department of Molecular Physiology and Biophysics, Baylor College of Medicine, Houston, Texas 77030, USA; 2 Graduate Program in Molecular Physiology and Biophysics, Baylor College of Medicine, Houston, Texas 77030, USA; 3 Department of Chemistry, Rice University, Houston, Texas 77005, USA; 4 Graduate Program in Immunology, Baylor College of Medicine, Houston, Texas 77030, USA; 5 Graduate Program in Translational Biology and Molecular Medicine, Baylor College of Medicine, Houston, Texas 77030, USA; 6 Department of Medicine, Baylor College of Medicine, Houston, Texas 77030, USA; 7 Biology of Inflammation Center, Baylor College of Medicine, Houston, Texas 77030, USA; 8 The NanoCarbon Center, Rice University, Houston, Texas 77005, USA; 9 Center for Drug Discovery, Baylor College of Medicine, Houston, Texas 77030, USA

## Abstract

Autoimmune diseases mediated by a type of white blood cell—T lymphocytes—are currently treated using mainly broad-spectrum immunosuppressants that can lead to adverse side effects. Antioxidants represent an alternative approach for therapy of autoimmune disorders; however, dietary antioxidants are insufficient to play this role. Antioxidant carbon nanoparticles scavenge reactive oxygen species (ROS) with higher efficacy than dietary and endogenous antioxidants. Furthermore, the affinity of carbon nanoparticles for specific cell types represents an emerging tactic for cell-targeted therapy. Here, we report that nontoxic poly(ethylene glycol)-functionalized hydrophilic carbon clusters (PEG-HCCs), known scavengers of the ROS superoxide (O_2_^•−^) and hydroxyl radical, are preferentially internalized by T lymphocytes over other splenic immune cells. We use this selectivity to inhibit T cell activation without affecting major functions of macrophages, antigen-presenting cells that are crucial for T cell activation. We also demonstrate the *in vivo* effectiveness of PEG-HCCs in reducing T lymphocyte-mediated inflammation in delayed-type hypersensitivity and in experimental autoimmune encephalomyelitis, an animal model of multiple sclerosis. Our results suggest the preferential targeting of PEG-HCCs to T lymphocytes as a novel approach for T lymphocyte immunomodulation in autoimmune diseases without affecting other immune cells.

Autoimmune diseases are characterized by loss of tolerance of the immune system for auto-antigens and the subsequent damage to the body caused by its own immune cells. One type of immune cell, T lymphocytes, are important participants in the pathogenesis of a large number of autoimmune diseases^1^. While the etiology of autoimmunity is not fully understood, a variety of factors including genetic susceptibility and environment triggers, such as infections, can lead to the loss of self-tolerance by T cells and consequently their ability to distinguish self from non-self, causing these cells to target one’s own organs and tissues^2^. Multiple sclerosis (MS) is a classic example of one of the many tissue-specific chronic T cell-mediated autoimmune diseases. In MS, T cells are thought in many instances to be targeting myelin—the insulating cover of neurons in the brain and spinal cord—leading to neurodegeneration, a wide range of physical and mental symptoms, and shortened life span^3^. Many current therapeutics for autoimmune diseases function as broad-spectrum immunosuppressants that target a variety of immune cells or other mediators of inflammation. They share a common trait: the potential to induce a wide range of serious side effects including increased risk of life-threatening infections and cancer^4^,^5^.

Excessive production of reactive oxygen species (ROS) has been implicated in the pathogenesis of multiple T cell-mediated autoimmune diseases^4^,^6^,^7^,^8^,^9^. Indeed, the significance of ROS as a therapeutic target for MS has been highlighted with dimethyl fumarate, an approved therapeutic for this disease^10^. Dimethyl fumarate was first tested for efficacy in MS because of its ability to activate the nuclear factor E2-related factor 2 (Nrf2), a leucine zipper transcription factor, which in turn induces the transcription of antioxidant response element-driven genes and the production of an array of detoxifying antioxidant proteins^10^,^11^. While dimethyl fumarate is beneficial in MS, it induces the apoptosis of activated T cells, leading to deleterious lymphopenia and potent and broad immunosuppression in all immune cells^12^. In addition, Nrf2 levels decrease with age, suggesting a potential loss of Nrf2-mediated efficacy of dimethyl fumarate in older patients^13^. Finally, studies in Nrf2^−/−^ mice demonstrated that dimethyl fumarate affects immune cell functions in a Nrf2-independent manner^14^.

Endogenous and dietary antioxidants, such as vitamins C and E, have shown only modest clinical efficacy in autoimmunity^6^,^8^, likely due to their poor selectivity for radical annihilation, limited stoichiometric capacity, and dependence on detoxifying molecules^15^. Thus, these are not promising candidates for therapeutic intervention for autoimmune diseases. Moreover, antioxidant dietary supplements require the administration of high doses, which increases mortality, likely due to their indiscriminate effects on all ROS^16^. A more targeted approach to modulating specific ROS involved in the pathogenesis of autoimmune diseases is likely to display benefits with fewer side effects. Interestingly, low levels of intracellular, but not extracellular, superoxide radicals (O_2_^•−^), which are ROS produced by the mitochondria in response to T cell receptor engagement during T cell activation, act as a crucial second messenger during T lymphocyte activation^17^,^18^,^19^,^20^,^21^. Since most current therapies for autoimmune diseases are broad-spectrum immunosuppressants associated with deleterious side effects^4^,^5^, intracellular O_2_^•−^ represents an attractive target for modulating T cell activity.

Functionalized carbon nanomaterials, such as fullerenes, single-walled and multiwalled carbon nanotubes, exhibit antioxidant properties superior to those of dietary antioxidants and have been used in a wide range of medical applications including preclinical studies of inflammatory arthritis and neurodegenerative diseases^15^,^22^. Carbon nanomaterials have also demonstrated remarkable affinity towards particular cell types and consequently have been used as shuttles for targeted drug delivery^23^. A major challenge precluding the translation of carbon nanomaterials into the clinic has been their associated cellular toxicity^24^. However, nanomaterials that are hydrophilic, have no trace metals and that do not form fibrous aggregates, are associated with little to no toxicity^15^. One such example is poly(ethylene glycol)-functionalized hydrophilic carbon clusters (PEG-HCCs), which have been used as both as a nanovector^25^ and as an antioxidant in the context of traumatic brain injury^15^. Indeed, high doses of PEG-HCCs have induced no acute toxicity involving the heart, liver, kidney, spleen or brain in extended mouse studies^25^. In addition, PEG-HCCs possess clear benefits over currently available antioxidants by specifically and more effectively scavenging O_2_^•−^ and its derivative, hydroxyl radical^15^,^23^,^25^,^26^. Interestingly, PEG-HCCs accumulate in the spleen of rodents^25^, a secondary lymphoid organ. Consequently, here, we determined the ability of splenic immune cells to internalize PEG-HCCs and the ability of the nanoparticles to alter immune cell function and inflammation, *ex vivo* and *in vivo*.

## Results

### PEG-HCCs are preferentially internalized by T lymphocytes over other splenic immune cells

**Figure 1:**
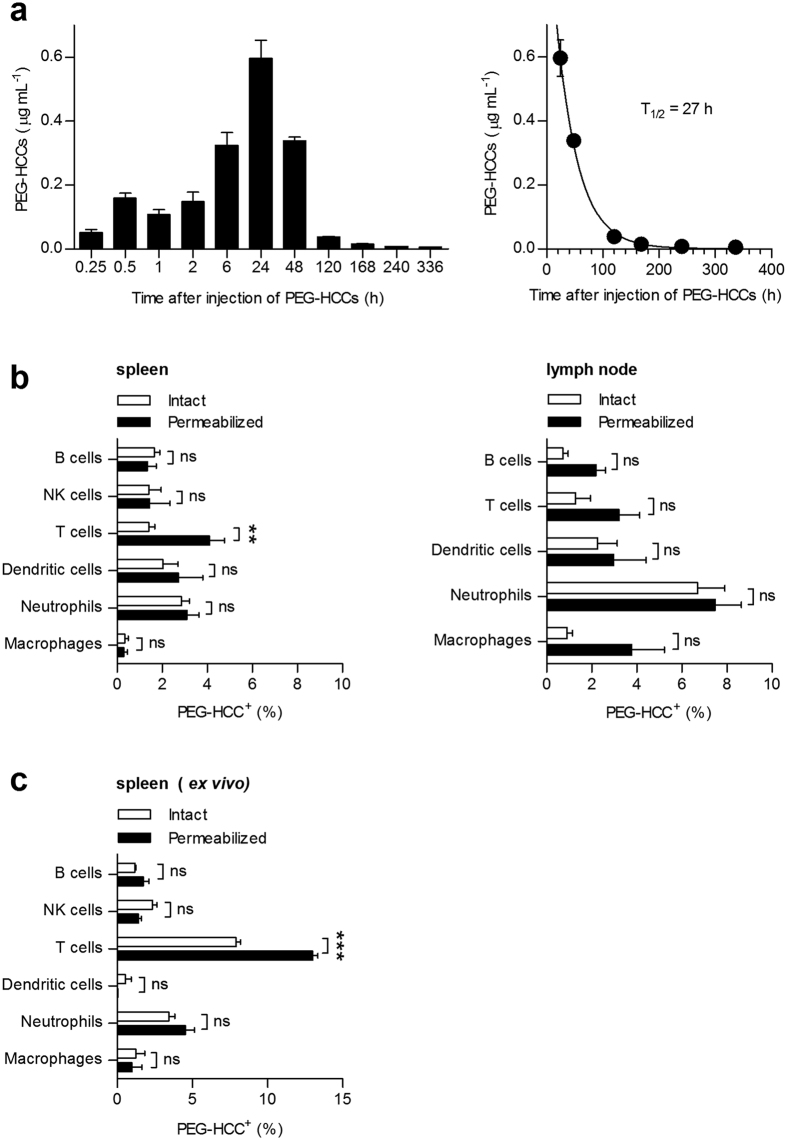
PEG-HCCs are preferentially internalized by T lymphocytes. (**a**) PEG-HCC levels in rat serum determined by anti-PEG ELISA after a single subcutaneous injection of 2 mg/kg body weight PEG-HCCs (left) (*n* = 5 rats/time-point). Data fit to a single exponential decay to calculate circulating half-life of 27 h (right). (**b**) FCM quantification of immune cell uptake of PEG-HCCs from spleen (left, *n* = 9 rats) and lymph nodes (right, *n* = 6 rats), collected 24 h after subcutaneous injection. The increased PEG signal after cell permeabilization is indicative of nanoparticle internalization; analyzed with two-way ANOVA. (**c**) Quantification of FCM data showing preferential uptake of PEG-HCCs by T cells over other splenic immune cells *ex vivo* (*n* = 3 spleens, two-way ANOVA). Mean ± s.e.m. **P < 0.01, ***P < 0.001.

As PEG-HCCs were detectable in the spleens of rodents following systemic administration^25^, we investigated whether splenic immune cells internalize PEG-HCCs *in vivo*. We first verified that PEG-HCCs contain acceptably low levels^27^ of endotoxins (Supplementary Table S1). We then determined their pharmacokinetics in rats by performing an enzyme-linked immunosorbent assay (ELISA) on serum samples collected after a single subcutaneous injection of 2 mg/kg in the scruff of the neck, a dose previously shown effective in rat studies of traumatic brain injury^15^. Maximum circulating levels of PEG-HCCs (0.6 μg/mL) were reached 24 h after injection (Fig. 1a), likely due to the formation of a subcutaneous slow-release depot, previously observed with other hydrophilic compounds^28^. The circulating half-life following this peak was 27 h, higher than previously reported after intravenous delivery of PEG-HCCs^25^, again likely due to a slow release of the nanoparticles from the site of injection^28^. These data demonstrate subcutaneous bioavailability and sufficient circulating levels of PEG-HCCs for *in vivo* use in rats. Based on our pharmacokinetic results, we injected rats subcutaneously with 2 mg/kg PEG-HCCs; 24 h later, at the peak of circulation levels, we prepared single-cell suspensions from the spleen, thymus, and inguinal lymph nodes draining the injection site (Supplementary Fig. S1). Cells were then washed, labeled with antibodies to cell surface markers of immune cells, fixed, and incubated with anti-PEG antibodies to detect the nanoparticles by multi-parametric flow cytometry (FCM) (Supplementary Figs S1 and S2). Cells were either left intact before anti-PEG staining to detect PEG-HCCs at the cell surface, or they were permeabilized before addition of the anti-PEG antibodies to detect PEG-HCCs both at the cell surface and inside the cells (Supplementary Fig. S1). We obtained a stronger signal from the anti-PEG antibodies after permeabilization of splenic T lymphocytes (Fig. 1b, left), demonstrating internalization of the nanoparticles by these cells. In contrast, macrophages, neutrophils, B lymphocytes, and natural killer lymphocytes in the spleen showed no difference in PEG-HCCs staining whether the cells were permeabilized or not prior to incubation with anti-PEG antibodies, showing that these immune cells do not internalize PEG-HCCs. No internalization of PEG-HCCs was detected in dendritic cells but the sample size for this population was relatively low in rat splenic preparations compared to the other cell types analyzed (Supplementary Fig. S2b). This result demonstrates that *in vivo*, PEG-HCCs are preferentially internalized by T cells over other splenic immune cells. This is in sharp contrast to results obtained with other nanoparticles that are internalized by phagocytic cells, such as macrophages or dendritic cells, rather than lymphocytes^29^,^30^,^31^,^32^. Data were confirmed in the spleen by immunohistochemistry using paraffin-embedded sections from spleens collected 24 h after subcutaneous administration of PEG-HCCs and double-stained for CD3, a marker of T cells, and PEG (Supplementary Fig. S3). PEG-HCCs were not internalized by immune cells in the inguinal lymph nodes (Fig. 1b, right), although they could be observed in the intact tissue (Supplementary Fig. S4), suggesting PEG-HCCs may not remain in the lymph nodes sufficiently long enough to be internalized by T cells, unlike in the spleen, a known site of PEG-HCC accumulation^25^. Interestingly, India ink (carbon black), which has been used extensively as a tracer for lymphatics, also flows through lymph nodes. By histology, India ink is only detected in the lymphatics and inside macrophages in the lymph nodes of rodents, not in the B or T lymphocyte zones^33^. Our data suggest that PEG-HCCs follow a similar path through lymphatics after subcutaneous injection but are not internalized by macrophages and are not in contact long enough with T lymphocytes for detectable internalization. Furthermore, PEG-HCCs could not be detected in the thymus, an organ crucial for T lymphocyte maturation, suggesting that T cell maturation will not be affected by the nanoparticles. We next determined whether this *in vivo* selectivity of PEG-HCCs for T lymphocytes over other splenic immune cells is maintained *ex vivo*. Based on the maximal levels reached in rat serum by the nanoparticles after a single injection of 2 mg/kg, we incubated rat splenocytes *ex vivo* with 0.1 μg/mL PEG-HCCs and found that, similar to our *in vivo* findings, the nanoparticles were detectable inside T cells but not other splenic immune cells (Fig. 1c).

To demonstrate the reliability of our FCM technique for PEG-HCC detection, we used thermogravimetric analysis to show that the PEG moieties remain attached to HCCs in the incubation conditions used in our assays (Supplementary Fig. S5). In addition, we performed the flow cytometric analysis with splenocytes incubated in the absence of PEG-HCCs or labeled with secondary antibodies in the absence of primary anti-PEG antibodies, showing minimal positive staining and therefore verifying selectivity of the detection (Supplementary Fig. S6). Similarly, a PEG signal was not detected in spleen sections from rats that had received vehicle instead of PEG-HCCs (Supplementary Fig. S3). Finally, nanoparticles were not detectable in dead T cells, which are readily permeable, thus precluding a non-specific uptake mechanism (Supplementary Fig. S7).

### Kinetics of PEG-HCC entry and exit from T cells

**Figure 2:**
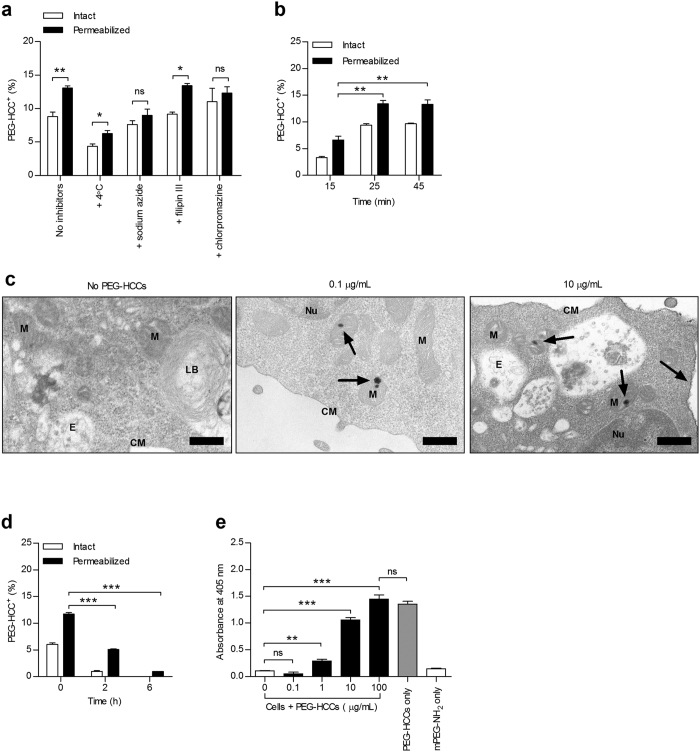
PEG-HCCs are internalized by T lymphocytes via clathrin-mediated endocytosis and do not accumulate inside the cells. (**a**) Rat splenic T cell uptake of PEG-HCCs, analyzed by FCM, at 37 °C or 4 °C and after incubation with sodium azide, filipin III, or chlorpromazine (*n* = 4 spleen preparations; 4000 live T cell singlets analyzed per sample). (**b**) Kinetics of nanoparticle internalization in splenic T cells incubated for the indicated times with 0.1 μg/mL PEG-HCCs prior to FCM (*n* = 3 spleen preparations, two-tailed Student’s *t* test). (**c**) Subcellular localization of PEG-HCCs in T cells, visualized by transmission electron microscopy, loaded with no PEG-HCCs (left) or a low (0.1 μg/mL, middle) or high (10 μg/mL, right) dose of PEG-HCCs. Arrows point to bundles of PEG-HCCs. Nucleus (Nu); mitochondrion (M); cell membrane (CM); lamellar body (LB); endosome (E). Scale bars, 400 nm. See Supplementary Fig. S10 for more images. (**d**) Kinetics of nanoparticle loss in splenic T cells incubated for 30 min with 0.1 μg/mL PEG-HCCs, washed, and analyzed by FCM after the indicated times (*n* = 3 spleen preparations, two-tailed Student’s *t* test). (**e**) PEG-HCC exit from splenic T cells measured by a cell-based sandwich ELISA (*n* = 3 experiments, one-way ANOVA). Mean ± s.e.m. *P < 0.05, **P < 0.01, ***P < 0.001.

To determine if PEG-HCCs enter T cells via active processes, we incubated cells at 4 °C or with sodium azide and found that both conditions attenuated nanoparticle internalization. Chlorpromazine, an inhibitor of clathrin-mediated endocytosis abrogated PEG-HCC internalization, unlike filipin II, an inhibitor of caveolin-mediated endocytosis, thus demonstrating that T lymphocytes internalize PEG-HCCs via clathrin-mediated endocytosis (Fig. 2a). Next, we examined the kinetics of PEG-HCC uptake by T lymphocytes and found maximal intracellular levels after a 25 min incubation at 37 °C; increasing incubation time did not further increase internalization levels (Fig. 2b and Supplementary Fig. S8). We then used transmission electron microscopy to probe the subcellular localization of PEG-HCCs (0.1 μg/mL) in T cells and observed bundles of the nanoparticles in mitochondria. At higher doses (10 μg/mL), PEG-HCCs were also visualized at the cell membrane (Fig. 2c and larger images in Supplementary Fig. S9).

Accumulation of nanomaterials in cells would increase the risk for toxicity; we therefore assessed the ability of T cells to release or degrade the PEG-HCCs. When T lymphocytes were incubated with PEG-HCCs for 30 min to allow internalization, washed, and maintained in media at 37 °C, both external and internal levels of PEG-HCCs decreased over 6 h (Fig. 2d and Supplementary Fig. S8), demonstrating that the nanoparticles do not accumulate inside the T cells over time. To determine whether this loss in detection is due to rapid degradation or exit from the cells, we performed a cell-based sandwich ELISA (Supplementary Fig. S10). In the absence of cells, PEG-HCCs were readily detected and saturated the detection signal (Fig. 2e). When T lymphocytes loaded with increasing concentrations of PEG-HCCs were placed onto the antibody-coated plate for 5 h, PEG-HCCs were detected in a dose-dependent manner, indicating that PEG-HCCs exit the T lymphocytes. Importantly, unconjugated mPEG-NH_2_ (5000 kDa) alone was not detected by the assay, providing further confirmation that the anti-PEG antibodies used throughout this study can only detect conjugated PEG moieties as claimed by its manufacturer.

### PEG-HCC internalization is not biased towards major T cell subsets

**Figure 3:**
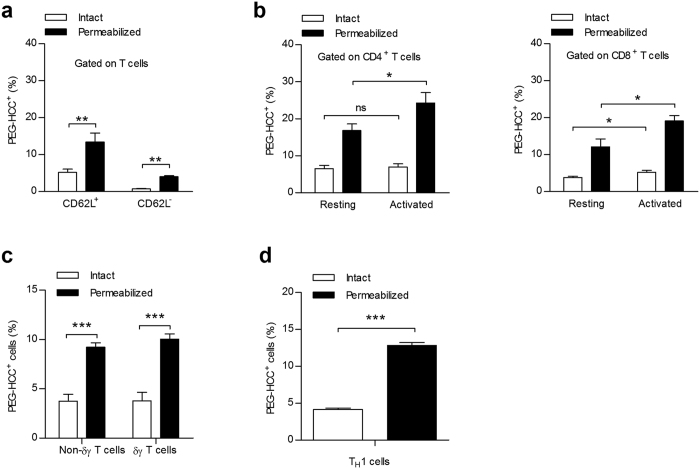
PEG-HCCs internalization is improved in activated T cells but is not biased towards major T cell subsets. (**a**) PEG-HCC internalization in naïve (CD3^+^CD62L^+^) and memory (CD3^+^CD62L^−^) rat splenic T cells, analyzed by FCM (*n* = 3 splenic preparations). (**b**) PEG-HCC internalization in helper (CD3^+^CD4^+^, left) and cytotoxic (CD3^+^CD8^+^, right) rat splenic T cells, analyzed by FCM (*n* = 3 splenic preparations). A separate group of rat splenocytes cells were also stimulated with the mitogen, concanavalin A (1 μg/mL). (**c**) PEG-HCC internalization in the δγ T cell subset (CD3^+^δγ^+^) as compared to non-δγ T cells (CD3^+^δγ^−^) from rat splenocytes, analyzed by FCM (*n* = 3 splenic preparations). (**d**) PEG-HCC internalization in primary CD3^+^CD4^+^, T_H_1-polarized effector memory rat T cells, analyzed by FCM (*n* = 3 splenic preparations). Mean ± s.e.m. *P < 0.05, **P < 0.01, ***P < 0.001.

Since PEG-HCCS are not internalized by all T cells, we used FCM to determine whether the nanoparticles are preferentially targeted to specific T lymphocyte subsets. We began our analysis with two major subpopulations of T cells: naïve (CD62L^+^) and memory (CD62L^−^) T cells (Fig. 3a), and helper (CD4^+^) and cytotoxic (CD8^+^) T cells (Fig. 3b); all of which internalize PEG-HCCs to a similar extent. Next, we determined whether PEG-HCCs distinguish between resting T cells and T cells activated with a mitogen; T lymphocyte activation improved nanoparticle uptake, although resting T lymphocytes could internalize them too (Fig. 3b). As our data had indicated that PEG-HCCs entered approximately 5–10% of T cells, we investigated T cell subsets present at a lower frequency in the spleen. We first analyzed δγ T cells that represent a small fraction of all T cells and also which play a role in autoimmunity^34^, but found no difference between these and other T cells in terms of PEG-HCC internalization (Fig. 3c). Additionally, we studied primary T helper 1 (T_H_1)-polarized effector memory T cells, a population implicated in some autoimmune diseases^35^, and found that PEG-HCCs were also internalized by these cells to a similar extent as other T cells (Fig. 3d). Taken together, these findings suggest that, while PEG-HCCs are preferentially internalized by T cells over other splenic immune cells, they do not target a specific subset of T cells.

### PEG-HCCs inhibit the activation of rat and human T lymphocytes

**Figure 4:**
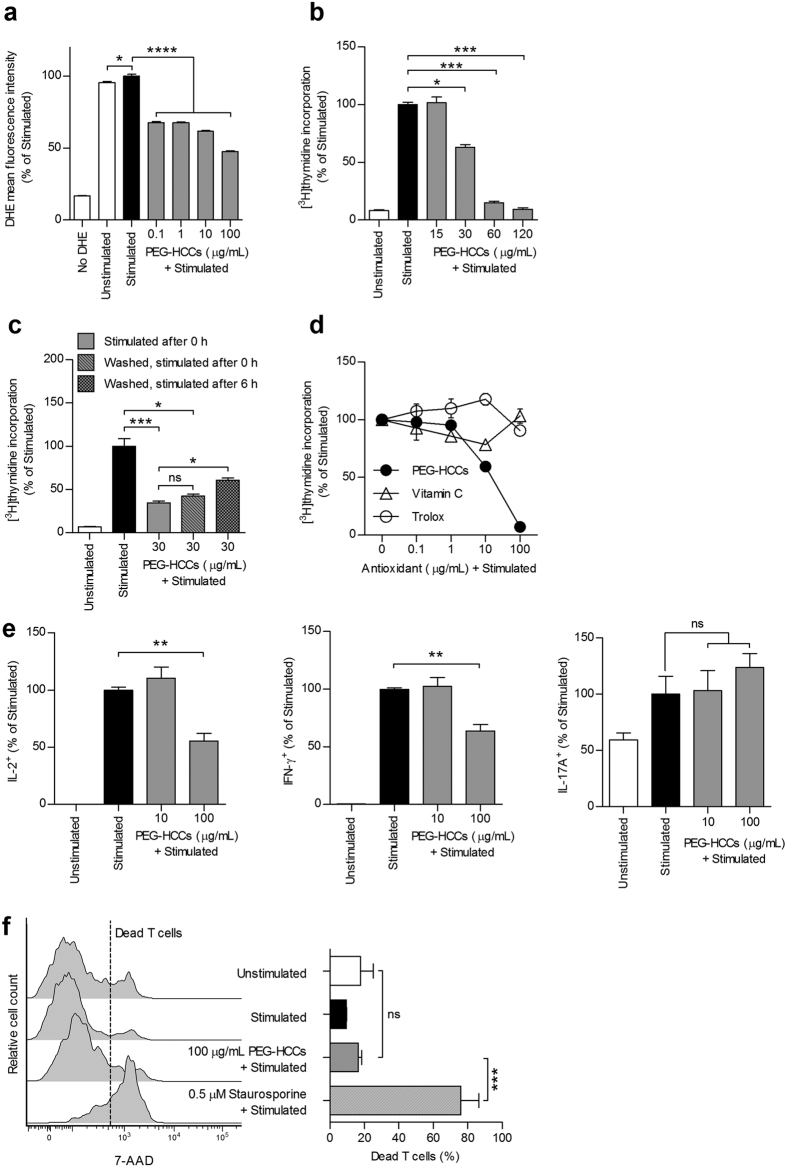
PEG-HCCs scavenge intracellular O_2_^•−^ and suppress T lymphocyte activity. (**a**), Detection of O_2_^•−^ levels by DHE fluorescence and FCM in primary ovalbumin-specific rat T cells. Cells were stained with dihydroethidium (DHE), incubated with the indicated concentrations of PEG-HCCs, and stimulated with phorbol 12-myristate-acetate (PMA) and ionomycin to induce intracellular O_2_^•−^ production. Unstained cells and unstimulated cells are shown as controls (*n* = 3 replicates, one-way ANOVA). (**b**) Proliferation of antigen-stimulated ovalbumin-specific T cells, measured by [^3^H] thymidine incorporation, in the presence of the indicated concentrations of PEG-HCCs. Unstimulated cells are shown as controls (*n* = 3 experiments). (**c**) Ovalbumin-specific T cells were incubated with PEG-HCCs for 30 min. Cells were then either antigen-stimulated without washing PEG-HCCs away (stimulated after 0 h), or washed to remove excess PEG-HCCs and stimulated immediately (washed, stimulated after 0 h) or 6 h later (washed, stimulated after 6 h) for the measurement of T cell proliferation. Unstimulated cells are shown as controls (*n* = 3 experiments). (**d**) Comparison of PEG-HCCs with vitamin C and trolox on the proliferation of antigen-stimulated ovalbumin-specific T cells (*n* = 3 replicates). (**e**) Detection by FCM of intracellular proinflammatory cytokines IL-2 (left), IFN-γ (middle) in antigen-stimulated GFP-transduced T cells, and IL-17A (right) in lymph node T cells incubated with the indicated concentrations of PEG-HCCs. Unstimulated cells are shown as controls (*n* = 3 experiments). (**f**) Quantification of cell death by FCM in T cells left unstimulated, antigen-stimulated, incubated with PEG-HCCs prior to antigen-stimulation, or antigen-stimulated and treated with staurosporine. Left, representative FCM histograms. Right, data quantification (*n* = 3 replicates, one-way ANOVA). Mean ± s.e.m. *P < 0.05, **P < 0.01, ***P < 0.001, ****P < 0.0001.

We assessed the consequences of PEG-HCC internalization on *ex vivo* T cell activity. PEG-HCCs induced a reduction in both intracellular O_2_^•−^ levels and proliferation of antigen-stimulated ovalbumin-specific or myelin basic protein-specific primary rat CD4^+^ T cells (Fig. 4a,b and Supplementary Fig. S11)^36^,^37^,^38^. Human peripheral blood T cells or rat splenic T cells (Supplementary Fig. S12) stimulated with mitogens were similarly sensitive to PEG-HCCs. In contrast, mPEG-NH_2_ (5000 kDa), used alone as a control, did not affect T cell proliferation (Supplementary Fig. S13), whereas non-PEGylated HCCs did inhibit proliferation (Supplementary Fig. S11). However, unlike PEG-HCCs, HCCs flocculate rapidly in water (Supplementary Fig. S11), precluding their *in vivo* use.

Washing away excess PEG-HCCs and then immediately stimulating the rat T cells did not alter their effect on proliferation (Fig. 4c). In contrast, stimulating the T cells 6 h after washout rescued the inhibitory effect on proliferation (Fig. 4c), demonstrating that the nanoparticles need to be internalized to decrease T lymphocyte activity. This result is in alignment with the kinetics shown in Fig. 2d and suggests that PEG-HCCs exert a reversible effect on T cell activity. We also compared the efficacy of PEG-HCCs with that of vitamin C and trolox (water-soluble analog of vitamin E) and found that these two well-characterized antioxidants had no effect on T lymphocyte proliferation (Fig. 4d), suggesting the higher antioxidant capacity of PEG-HCCs^26^ is necessary for this inhibitory effect. Finally, we used FCM to examine the effects of PEG-HCCs on the production of proinflammatory cytokines involved in autoimmune diseases, such as MS, by antigen- or mitogen- stimulated T cells and found a ~30% reduction in the intracellular levels of the T_H_1 cytokines interleukin (IL)-2 and interferon (IFN)-γ, but no effect on expression levels of the T_H_17 cytokine IL-17A (Fig. 4e and Supplementary Fig. S14), suggesting distinct cell signaling pathways are altered by PEG-HCCs.

Dimethyl fumarate induces the apoptosis of activated T cells^12^. To determine whether the observed effects of PEG-HCCs on T cell proliferation and cytokine production were due to nanoparticle cytotoxicity, we used FCM to analyze cell death in ovalbumin-specific and myelin basic protein-specific rat T cells incubated with PEG-HCCs or HCCs prior to stimulation and found no changes in cell viability (Fig. 4f and Supplementary Fig. S11). In addition, treating unstimulated T cells with PEG-HCCs did not affect their basal homeostatic proliferation in the absence of antigen or exogenous cytokines (Supplementary Fig. S15), suggesting that an increase in intracellular O_2_^•−^ levels during T lymphocyte activation^17^,^18^,^19^,^20^,^21^ is necessary for PEG-HCCs to alter cellular function.

### PEG-HCCs leave key functions of macrophages unaltered

**Figure 5:**
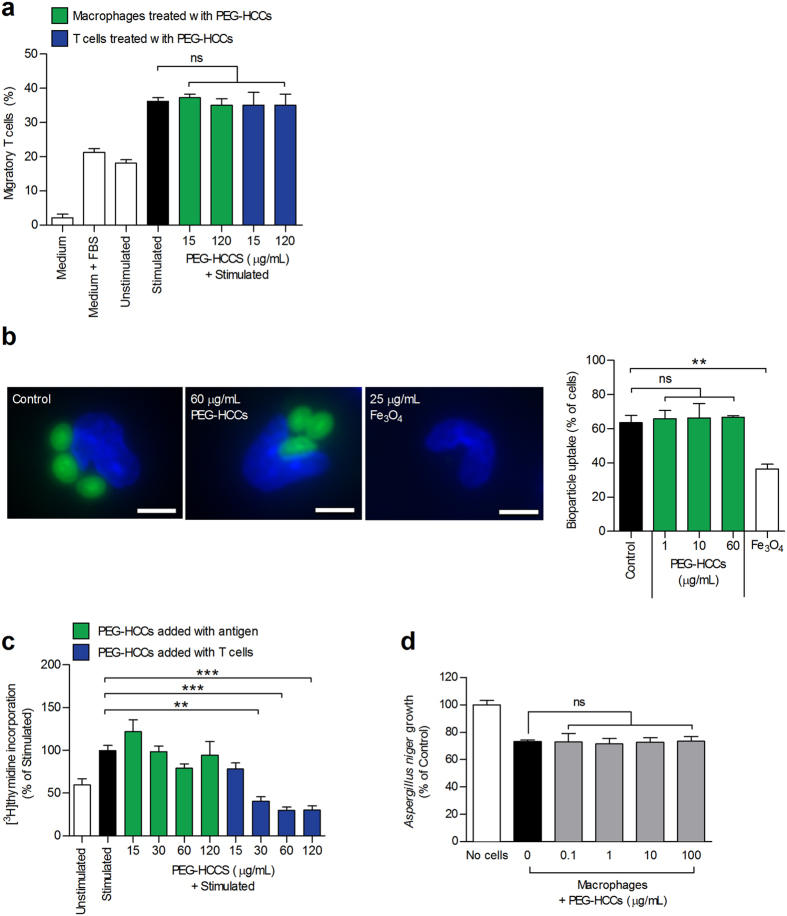
Inability to internalize PEG-HCCs leaves key macrophage functions unaltered. (**a**) Percentage of migratory ovalbumin-specific T cells through a 5 μm transwell filter when the bottom chamber contained supernatant from LPS-stimulated macrophage cultures, used as a readout for adequate macrophage production of T cell chemoattractants. Green bars: Macrophages incubated with PEG-HCCs during LPS-stimulation and T cells left untreated. Blue bars: Macrophages left untreated during LPS-stimulation and T cells incubated with PEG-HCCs and washed prior to the transwell assay. Bottom chambers containing medium alone, medium supplemented with 5% FBS, or supernatant from unstimulated macrophages are shown as controls (*n* = 3 replicates). (**b**) Left, representative images of macrophage phagocytosis of Alexa Fluor 488-conjugated bioparticles (green) in the absence of nanoparticles (control) or in the presence of PEG-HCCs or Fe_3_O_4_ nanoparticles. Macrophage nuclei were stained with DAPI (blue). Right, quantification of the phagocytosis assay (*n* = 100 cells/rat; N = 3 rats spleens, two-tailed Student’s *t* test). Scale bars, 5 μm. (**c**) Proliferation of ovalbumin-specific T cells stimulated in the presence of antigen-loaded macrophages, used to gauge adequate macrophage antigen processing and presentation. Green bars: Macrophages incubated with PEG-HCCs during antigen loading. Blue bars: PEG-HCCs added at same time as T cells. Macrophages not loaded with antigen (unstimulated) were used as a control (*n* = 3 experiments). (**d**) *Aspergillus niger* fungus growth prevention by rat splenic macrophages treated with the indicated doses of PEG-HCCs (*n* = 3 experiments). Mean ± s.e.m. *P < 0.05, **P < 0.01, ***P < 0.001.

We demonstrated in Fig. 1 that macrophages do not internalize PEG-HCCs *in vivo* or *ex vivo*. However, because macrophages critically regulate T cell function through a variety of mechanisms, including chemoattractant production, phagocytosis, and antigen processing and presentation, we determined whether PEG-HCCs influenced any of these macrophage activities. We stimulated primary rat splenic macrophages with lipopolysaccharide (LPS) to induce the secretion of T lymphocyte chemoattractants, as measured by the ability of macrophage culture supernatants to induce T cell migration through transwell membranes. Adding PEG-HCCs during macrophage stimulation did not affect T lymphocyte migration (Fig. 5a), demonstrating that the nanoparticles do not alter the production of T cell chemoattractants by macrophages. Additionally, incubating unstimulated T lymphocytes with PEG-HCCs did not alter their migration (Fig. 5a), in agreement with our finding that PEG-HCCs do not affect unstimulated T cells. We further found that, unlike other nanoparticles^39^, PEG-HCCs do not affect phagocytosis by macrophages (Fig. 5b and Supplementary Fig. S16). To measure antigen processing and presentation, macrophages were loaded with ovalbumin, washed, and provided ovalbumin-specific T cells; T cell proliferation was used to assess adequate antigen processing and presentation (Supplementary Fig. S17). T cell proliferation was unaffected when PEG-HCCs were added to macrophages upon ovalbumin-loading, demonstrating a lack of effects of PEG-HCCs on antigen processing and presentation by macrophages (Fig. 5c). However, the addition of PEG-HCCs to macrophages at the same time as the T cells led to a reduction in T cell proliferation (Fig. 5c), similar to our findings in Fig. 4b.

In addition to their role as antigen-presenting cells, macrophages play a central part in pathogen defense. We therefore tested the effects of PEG-HCCs on the ability of macrophages to prevent the growth of the fungus *Aspergillus niger* and found no fungistatic effects (Fig. 5d). As a control, we verified that PEG-HCCs alone do not induce fungistasis (Supplementary Fig. S18). These results indicate that PEG-HCCs do not alter major functions of macrophages, likely as a result of the inability of these cells to internalize the nanoparticles.

### PEG-HCCs are effective in reducing memory T lymphocyte reactions *in vivo*

**Figure 6:**
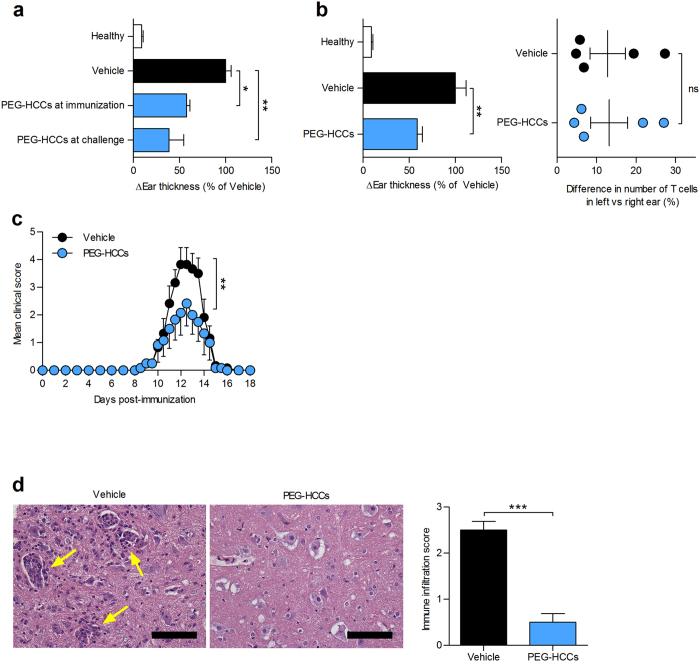
Administration of PEG-HCCs suppresses T lymphocyte–mediated inflammation and ameliorates experimental autoimmune encephalomyelitis. (**a**) An active DTH reaction was induced against ovalbumin in rats. Rats received either saline (vehicle) or PEG-HCCs (2 mg/kg body weight, subcutaneous) at time of immunization or challenge. Difference in ear thicknesses for each rat was measured 48 h after challenge. Healthy unimmunized rats are shown as a control (*n* = 5 rats per group). (**b**) Adoptive DTH induced in rats via adoptive transfer of antigen-activated GFP-transduced ovalbumin specific primary rat T cells. Treatment and ear measurements (left) identical to a. Detection of GFP-transduced ovalbumin-specific T cells in ears using FCM (right). Healthy rats that did not receive adoptive transfer or challenge are shown as a control (*n* = 10 rats in vehicle group; 9 rats in PEG-HCC-treated group). (**c**) Clinical scores of rats with acute active EAE, treated with saline (vehicle) or 2 mg/kg PEG-HCCs subcutaneously every 3 days, beginning at onset of disease signs (*n* = 6 rats per group). (**d**) Histological analysis of spinal cord gray matter collected from rats with EAE at the peak of disease (day 12 post-immunization) and stained with hematoxylin and eosin. Left, representative images. Arrows point to immune infiltrates. Scale bars, 100 μm. Right, quantification of degree of immune infiltration from eight random fields of view (*n* = 3 rats per group). Mean ± s.e.m. *P < 0.05, **P < 0.01, ***P < 0.001.

The intriguing preferential immunomodulatory properties of PEG-HCCs on T lymphocytes over other splenic immune cells encouraged us to examine their effects in T lymphocyte-mediated inflammation. We chose delayed-type hypersensitivity (DTH), also known as type IV hypersensitivity, as a first model as it is a well-characterized model of inflammation mediated by memory T lymphocytes that have had previous encounter with their antigen. We elicited an active DTH response against ovalbumin in rats^40^ and found that a single subcutaneous injection of 2 mg/kg PEG-HCCs, at either time of immunization or challenge, was sufficient to decrease inflammation (Fig. 6a). A similar result was obtained in the adoptive model of DTH elicited by the injection of antigen-stimulated GFP-transduced ovalbumin-specific T cells, followed by ovalbumin challenge (Fig. 6b)^38^,^41^. Detection of the GFP-transduced ovalbumin-specific T cells by FCM at the site of inflammation showed no difference in homing of the T cells after vehicle or PEG-HCC treatment (Fig. 6b). This result suggests that the effects of PEG-HCCs on DTH are due to impaired T cell reactivation *in situ*, as found with some T lymphocyte-directed immunomodulators^38^, rather than homing inhibition as seen with other T cell immunomodulators^42^.

These beneficial effects of PEG-HCCs on active and adoptive DTH prompted us to test the effects of PEG-HCCs in rats with active acute experimental autoimmune encephalomyelitis (EAE), a model of MS. Subcutaneous treatment with 2 mg/kg PEG-HCCs every three days (equivalent to three circulating half-lives based on our pharmacokinetics data), starting after the onset of clinical signs, reduced disease severity (Fig. 6c, Supplementary Movie S1 and Movie S2). Histologic analysis of spinal cords collected from EAE rats at peak of disease (day 12 post-immunization) revealed a decrease in inflammatory foci (Fig. 6d), suggesting that PEG-HCCs reduce immune cell infiltration into the central nervous system.

## Discussion

In summary, we have demonstrated that the antioxidant carbon nanoparticles, PEG-HCCs, are immunomodulators that preferentially target T lymphocytes. We established that, unlike other nanoparticles that mainly target phagocytic cells^17^,^18^,^19^,^20^,^21^, PEG-HCCs are preferentially internalized by T lymphocytes over other immune cells. While we were not able to identify a single mechanism responsible for this preferential uptake, our data indicate that PEG-HCCs enter the cells mainly via clathrin-mediated endocytosis, similar to other carbon nanoparticles^43^. We took advantage of the unique property of PEG-HCCs to favor T cells to demonstrate that PEG-HCCs scavenge intracellular O_2_^•−^ produced via antigen-stimulation of T cells and, consequently, reversibly inhibit proliferation and the production of proinflammatory cytokines without cytotoxicity. Our findings are in line with studies demonstrating the use of antioxidants to attenuate T cell activation induced by mitogens or antigens^21^. We established that the inhibition of T cell activity by PEG-HCCs was not due to an extraneous effect on chemoattraction, phagocytosis, or antigen processing and presentation by macrophages, which are essential steps utilized by antigen-presenting cells for the physiological activation of T cells^44^.

A major implication of these data is that the inability to internalize PEG-HCCs allows key immune functions of macrophages to remain unaltered. This demonstrates that PEG-HCCs are selective for some immune cells and suggests that pathogen defense by macrophages remains intact. However, additional work is required to elucidate the effects of PEG-HCCs on T lymphocyte subpopulations that help to maintain self-tolerance such as regulatory T cells^45^. Furthermore, future studies will be necessary to determine if PEG-HCCs affect the use of O_2_^•−^ as a microbicidal agent by neutrophils^46^. However, the significance of our *ex vivo* results on T cell activity by PEG-HCCs was clearly demonstrated by the findings that administration of these nanoparticles into rat models led to a reduction in DTH response, immune infiltration into the spinal cord, and EAE scores. Together, these data suggest that PEG-HCCs are attractive new tools for treating T cell-mediated autoimmune diseases.

## Methods

### Animals

Female inbred Lewis rats were purchased from Charles River and group-housed in autoclaved cages with irradiated rodent chow and water *ad libitum* in a facility approved by the American Association for Laboratory Animal Science. Thymus donors were 38–42 days old. All remaining studies used rats aged 43–48 days old. All procedures were in accordance with National Institutes of Health guidelines and approved by the Institutional Animal Care and Use Committee at Baylor College of Medicine. Animals were euthanized following guidelines from the American Veterinary Medical Association.

### Cells

Splenocytes were isolated from spleens of naïve rats^47^. Mononuclear splenocytes were isolated using Histopaque-1077 (Sigma-Aldrich)^48^. Human peripheral blood cells were isolated from buffy coats (Gulf Coast Regional Blood Center, Houston, TX). Primary Lewis rat GFP-transduced ovalbumin-specific T cells were a kind gift from Alexander Flügel (Munich, Germany)^37^. Primary Lewis rat myelin basic protein-specific T cells were a kind gift from Evelyne Béraud (Université de la Méditerranée, Marseilles, France)^36^. As antigen-presenting cells for ovalbumin-specific or myelin-basic protein-specific T cell stimulation, either rat splenic macrophages or irradiated (30 Gy) rat thymocytes were used^47^. Lymph node cells were isolated from inguinal lymph nodes of rats with EAE at the peak of disease (day 12 post-immunization) by preparing a single-cell suspension using 70 μm cell strainers. Cells were cultured as described^49^ unless specified otherwise.

### Nanoparticles

For all experiments, PEG-HCCs and HCCs were prepared as described^25^. The materials were purified by size using crossflow (tangential) filtration. All samples were sterile-filtered before storage and further use. The starting source of carbon has remained unchanged since the first publication on PEG-HCCs^25^. PEG-HCCs are stable for >5 months in solution at neutral pH. Acid (pH < 2) will cause amide bond cleavage over long times at room temperature, and base (pH > 10) will cause further fragmentation of HCCs over time. However, there are no changes in Raman, IR or XPS over months in neutral water^50^. Endotoxin levels in PEG-HCCs were measured using ToxinSensor Chromogenic LAL Endotoxin Assay Kit (GenScript) using the manufacturer’s protocol.

### Pharmacokinetics

Blood was drawn^51^ at various time points after a single subcutaneous injection of 2 mg/kg body weight of PEG-HCCs in the scruff of the neck. Serum levels of PEG-HCCs were quantitated using a PEGylated protein ELISA kit (Enzo Life Sciences). Circulating half-life was determined by fitting the data to a single exponential decay.

### Flow cytometry

Cells were washed with ice-cold FCM buffer (PBS + 2% goat serum + 2% BSA), stained with antibodies (Table S2) and fixed with ice-cold PBS + 1% paraformaldehyde. Data were acquired on a FACSCanto II or LSRFortessa (Becton Dickinson) with the FACSDiva software within the Cytometry and Cell Sorting core at Baylor College of Medicine, and analyzed using FlowJo software (Treestar). For each sample, doublet discrimination was performed and 30,000 events were acquired.

### Cellular uptake of PEG-HCCs

Freshly isolated rat splenocytes were resuspended (1 × 10^6^ cells/mL) in medium + 5% FBS and incubated with or without PEG-HCCs (0.1 μg/mL) for the indicated times at 37 °C, 5% CO_2_ unless indicated in the figure caption. Cells were then washed with FCM buffer and incubated with purified mouse IgG (Life Technologies) to block FcγII/FcγIII receptors. Cells were stained with antibodies (Table S2) directed against surface markers, washed, and fixed. Cells were either left intact or permeabilized with freshly prepared 0.5% saponin (EMD Millipore) in FCM buffer, and then stained for PEG-HCCs with an anti-PEG antibody and analyzed by FCM (Supplementary Figs S1 and S2). Saponin was selected as the detergent as Tween-20 and Triton X-100 contain PEG and can produce false positive signals. 25 mM sodium azide (Sigma-Aldrich) was used to inhibit active transport processes; 1 μg/mL filipin III (Cayman Chemical) was used to block caveolin-mediated endocytosis; 10 μg/mL chlorpromazine (Sigma-Aldrich) was used to inhibit clathrin-mediated endocytosis^52^. Background signal from the anti-PEG antibody in the absence of PEG-HCCs was subtracted during analysis.

### Immunohistochemistry

Rats were injected with 2 mg/kg PEG-HCCs subcutaneously, humanely euthanized after 24 h and paraffin-embedded spleen sections were prepared. Sections were dewaxed with xylene (Fisher), hydrated through a gradient of ethanol, and washed in PBS. Antigen-recovery was performed using pre-heated sodium citrate buffer (10 mM, pH 6.5) for 15 min in microwave. Sections were cooled to room temperature and non-specific staining was prevented by incubating sections overnight in PBS + 5% goat serum + 5% BSA. Sections were incubated with anti-CD3 and anti-PEG antibodies for 2 h at room temperature, washed with PBS, and incubated with secondary conjugate antibodies for 1 h at room temperature. Sections were then washed and dehydrated in 100% ethanol through a gradient, soaked with xylene and mounted with SlowFade mountant with DAPI. Representative photos were taken at 10× magnification on an Olympus IX71 epifluorescence microscope equipped with an Olympus Qcolor 5 camera and the SimplePCI software, v.6.6.0.0^53^.

### Thermogravimetric analysis

Two mL of 1 mg/mL PEG-HCCs were incubated for 0, 1, and 24 h at 37 °C with 2 mL of fresh Lewis rat serum passed through a 100,000 MWCO filter (Amicon Ultra-4 Centrifugal Filter Unit). PEG-HCCs were isolated from serum by filtration through a second 100,000 MWCO filter and washed with 4 mL of deionized water three times. Remaining water was removed from PEG-HCCs by evaporation under reduced pressure and analyzed by thermogravimetric analysis (TA Instruments Q-600 Simultaneous TGA/DSC) to determine the mass percentages of PEG in the PEG-HCCs.

### Transmission electron microscopy

Ovalbumin-specific rat T cells (2 × 10^6^ cells) were resuspended in medium + 5% FBS and incubated with or without PEG-HCCs (0.1 or 10 μg/mL) for 30 min at 37 °C, 5% CO_2_. Cells were washed with PBS and pelleted by centrifuge for processing and imaging by the Integrated Microscopy Core at Baylor College of Medicine. Four separate pellets were dispersed in ice-cold modified Karnovsky’s fixative in 0.1 M cacodylate buffer at pH 7.37 for 2.5 hours. The suspension was re-pelleted at 6000 RPM for 10 min, supernatant decanted, and cells quenched in 0.4% FSG for 25 min. Pellets remained fixed to the side of 1.5 mL tubes and were processed in the tube for the remainder of the protocol. After 3 × 5 min washes with 0.1 M cacodylate buffer, the pellets were osmicated for 45 min and then held overnight in 0.1 M cacodylate buffer. The following morning, pellets were rinsed in 4 × 5 min ddH_2_O and dehydrated in 100% ethanol through a gradient series of ethanol. Pellets were gradually infiltrated through a gradient series of plastic-ethanol mixtures and left to infiltrate overnight in 100% plastic without catalyst. This was followed by several changes in pure plastic with catalyst, then pellets were embedded in Spurr Low Viscosity resin and polymerized at 62 °C for 3 days. Ultra-thin sections were cut on a Diatome Ultra45 knife using a Leica U6 ultramicrotome. Sections were collected on 150 hex-mesh copper grids, stained with saturated aqueous uranyl acetate and counter-stained with Reynold’s lead citrate. The stained sections were viewed on a Hitachi H7500 transmission electron microscope and images were captured using an AMT XR-16 digital camera and AMT Image Capture software, v602.600.51.

### Measurement of PEG-HCC exit from the T lymphocytes

Immulon 4 HBX flat-bottom 96-well plates were coated with purified anti-PEG antibodies (Table S2, 100 μl, 5 μg/mL) at 4 °C for 24 h. Resting ovalbumin-specific rat T cells were resuspended (1 × 10^6^ cells/mL) in medium + 5% FBS and incubated with or without PEG-HCCs (0.1–100 μg/mL) for 30 min at 37 °C, 5% CO_2_. Cells were washed thoroughly with medium while the antibody-containing plate was washed with PBS. Cells were seeded into the plate (1 × 10^5^ per well) and incubated for 5 h at 37 °C, 5% CO_2_. Cells were removed from the plate that was washed thoroughly with PBS. Biotinylated anti-PEG antibodies (Table S2) were added to wells (1 μg/mL), and incubated for 1 h at room temperature. The plate was washed thoroughly with PBS after which streptavidin conjugated to alkaline phosphatase was added to each well (1 μg/mL), and incubated for 20 min at room temperature. The plate was washed thoroughly and 1-step p-nitrophenyl phosphate (PNPP) substrate (ThermoScientific) was added to each well, incubated for 15–30 min at room temperature to allow color development, and 2 N sodium hydroxide was added to each well to stop the reaction. Absorbance was measured at 405 nm on a Multiscan EX plate reader (ThermoScientific).

### Superoxide measurement

Ovalbumin-specific rat T cells were resuspended in RPMI 1640 medium (1 × 10^6^ cells/mL) without supplements or serum, plated in a 6-well culture dish and loaded with 5 μM dihydroethidium (Life Technologies) for 30 min at 37 °C, 5% CO_2_. Cells were washed with RPMI 1640 and incubated with or without PEG-HCCs (0.1–100 μg/mL) for 30 min at 37 °C, 5% CO_2_. Cells were washed with PBS + 1% BSA and stimulated with 50 ng/mL PMA and 1 μg/mL ionomycin (Sigma-Aldrich) for 45 min prior to FCM analysis.

### T lymphocyte proliferation

Ovalbumin-specific or myelin basic protein-specific rat T cells were resuspended in medium + 5% FBS and seeded in a 96-well plate (5 × 10^4^ per well) in the presence of irradiated rat thymocytes (2 × 10^6^ per well), serving as antigen-presenting cells^38^,^54^,^55^,^56^. Rat mononuclear splenocytes and human peripheral blood cells were resuspended in medium + 5% FBS and seeded in a 96-well plate (1 × 10^5^ per well)^40^,^57^. Cells were incubated with or without antioxidants: trolox (Sigma-Aldrich), vitamin C (Sigma-Aldrich), PEG-HCCs, or HCCs for 30 min at 37 °C and 5% CO_2_. Ovalbumin-specific and myelin basic protein-specific rat T cells were stimulated with 10 μg/mL ovalbumin or guinea pig myelin basic protein (Sigma-Aldrich), respectively^36^,^38^,^58^. Rat mononuclear splenocytes and human peripheral blood cells were stimulated with 1 μg/mL concanavalin A or phytohemagglutinin, respectively^36^,^59^. Cells were cultured for 72 h at 37 °C and 5% CO_2_, and 1 μCi [^3^H] thymidine (MP Biomedicals) was added to each well during the final 16–18 h of incubation. Cells were then lysed by freezing and DNA was harvested on fiberglass filters using a cell harvester (Inotech Biosystems International). [^3^H] thymidine incorporation was measured on a β-scintillation counter (Beckman Coulter).

### Cytokine production by T lymphocytes

Ovalbumin-specific rat T cells and cells isolated from the draining lymph nodes of rats with EAE at peak of disease were resuspended (5 × 10^4^ cells/mL) in medium + 5% FBS and incubated with or without PEG-HCCs (10, 100 μg/mL) for 30 min at 37 °C and 5% CO_2_. Ovalbumin-specific T cells were stimulated by irradiated rat thymocytes (2 × 10^6^ cells/mL) pre-incubated with 10 μg/mL ovalbumin for 2 h. Lymph node cells were stimulated with 50 ng/mL PMA and 1 μg/mL ionomycin. To inhibit cytokine secretion, 10 μg/mL brefeldin A (eBioscience) was immediately added to all wells. Cells were then cultured 4 h at 37 °C and 5% CO_2_, fixed, and permeabilized with 0.1% Triton X-100 (Sigma-Aldrich). Ovalbumin-specific T cells were stained with anti-IL-2 and anti-IFN-γ antibodies, while lymph node cells were stained with an anti-IL-17A antibody, and appropriate secondary antibodies (Supplementary Table S2), and then analyzed by flow cytometry^59^.

### Antigen processing and presentation by macrophages

Freshly isolated rat splenic macrophages were resuspended in medium + 10% FBS, seeded in a 96-well plate (2 × 10^4^ per well), incubated with 10 μg/mL ovalbumin for 2 h to allow cells to adhere and process antigen, and then washed with PBS. Ovalbumin-specific rat T cells were resuspended in medium + 10% FBS and added to the macrophages (1 × 10^4^ per well). Macrophages were incubated with or without PEG-HCCs (15–120 μg/mL), for 30 min at 37 °C, 5% CO_2_, either at the same time as antigen loading or upon addition of T cells (Supplementary Fig. S18). An effect on macrophage antigen processing and presentation by PEG-HCCs was gauged by comparing T cell proliferation against vehicle-treated stimulated cells.

### Cytotoxicity assays

Cytotoxicity in response to PEG-HCCs was measured by FCM^60^. More details are provided in the Supplementary Information. Ovalbumin-specific and myelin basic protein-specific rat T cells were resuspended (1 × 10^6^ cells/mL) in medium + 5% FBS and incubated with or without PEG-HCCs or HCCs (100 μg/mL) for 30 min at 37 °C and 5% CO_2_ prior to stimulation with 10 μg/mL ovalbumin and myelin basic protein, respectively. Cells were cultured for 72 h at 37 °C and 5% CO_2_. Positive control wells received 0.5 μM staurosporine (EMD Millipore) during the last 16 h to induce cell death. Cells were then washed and stained with 7-aminoactinomycin D (Sigma-Aldrich) to detect dead cells, and analyzed by FCM.

### Macrophage chemokine production and T cell migration

Freshly isolated rat macrophages were resuspended (2 × 10^6^ cells/mL) in medium + 10% FBS and cultured overnight at 37 °C and 5% CO_2_ to allow macrophages to adhere. Cells were washed with PBS to remove non-adherent cells and incubated with or without PEG-HCCs (15, 120 μg/mL) for 30 min at 37 °C and 5% CO_2_. Macrophages were then stimulated with 10 ng/mL lipopolysaccharide (LPS)^61^. After 24 h incubation at 37 °C and 5% CO_2_, 0.6 mL supernatant was transferred to the bottom chamber of a 5 μm 24-well transwell filter plate (Costar) to serve as chemoattractant. Ovalbumin-specific T cells were resuspended (1 × 10^6^ cells/mL) in serum-free medium, incubated with or without PEG-HCCs (15, 120 μg/mL) for 30 min at 37 °C and 5% CO_2_, and 2 × 10^5^ cells were seeded in each transwell insert. As an input control, 2 × 10^5^ T cells were placed in the bottom chamber of one well without a transwell insert. The transwell plate was incubated at 37 °C and 5% CO_2_ for 4 h, prior to counting the bottom chambers for migratory T cells using a hemocytometer. Percent migratory cells was calculated using the following equation:  = [(number of cells in bottom chamber)/(number of cells in input control)] × 100. Chemoattractant containing either medium alone, medium + 10% FBS, or supernatant from unstimulated macrophages are shown as controls.

### Phagocytosis assays

Freshly isolated rat splenocytes (8 × 10^6^ cells/mL) were plated in chamber slides (Lab-Tek) for 2 h to allow macrophages to adhere. Slides were washed with PBS to remove non-adherent cells and remaining macrophages were incubated with or without PEG-HCCs (1–60 μg/mL) for 30 min at 37 °C, 5% CO_2_. Incubation with iron(II,III) oxide (Fe_3_O_4_) nanoparticles (25 μg/mL) served as a control for phagocytosis inhibition^39^. Alexa Fluor 488-conjugated zymosan A bioparticles (Life Technologies) were then added to macrophages following manufacturer’s instructions. Slides were incubated for 1 h at 37 °C and 5% CO_2_, fixed, and mounted with SlowFade with DAPI (Life Technologies). Representative photos were taken at 10× magnification with an Olympus IX71 epifluorescence microscope. Percent of phagocytizing cells was calculated in eight random fields of view using the following equation:  = [(number of macrophages with engulfed bioparticles)/(total number of macrophages)] × 100.

### Macrophage fungistatic assay

The ability of splenic macrophages to prevent growth of *Aspergillus niger* was tested as described^62^. Splenic macrophages were isolated by adhesion as described above and plated into 24 well plates (0.2 × 10^6^ cells/well) until adherent. Cells were treated with PEG-HCCs and *Aspergillus niger* (200 conidia/well) were added before a 24 h incubation at 37 °C, 5% CO_2_ and enumeration of hyphal growth.

### Delayed-type hypersensitivity induction and monitoring

For induction of active DTH, rats were immunized in the lower flank using ovalbumin emulsified in complete Freund’s adjuvant (Difco)^63^. For induction of adoptive DTH, GFP-transduced ovalbumin-specific rat T cells were activated *ex vivo* by stimulation with 10 μg/mL ovalbumin in the presence of irradiated rat thymocytes, serving as antigen-presenting cells^38^,^54^. Cells were incubated 48 h at 37 °C and 5% CO_2_ and then injected intraperitoneally into rats (1 × 10^6^ T cells/rat). Rats were randomly selected to receive a single subcutaneous injection in the scruff of the neck of saline (vehicle) or PEG-HCCs (2 mg/kg body weight) either at the time of immunization (active DTH only) or at time of challenge. One week after immunization or two days after adoptive transfer, animals were challenged with ovalbumin dissolved in PBS in the pinna of one ear and a sham injection of PBS in the opposite ear^38^,^54^. At 48 h post-challenge, ear swelling was measured using a spring-loaded micrometer (Mitutoyo) and the difference in ear thickness was calculated for each animal. Rats with adoptive DTH were humanely euthanized immediately after DTH measurement. After cardiac perfusion with saline, ears were collected and single-cell suspensions were prepared using a 70 μm cell strainer prior to flow cytometry analysis to detect GFP transduced cells at the site of inflammation^38^.

### Acute active EAE induction and histology

EAE was induced by immunizing rats with an emulsion of guinea pig myelin basic protein (Sigma-Aldrich) in complete Freund’s adjuvant supplemented to a final concentration of 4 mg/mL heat-killed *Mycobacterium tuberculosis* H37Ra (Difco) at the base of the tail. Rats were randomly selected to receive subcutaneous treatment with saline (Vehicle) or PEG-HCCs (2 mg/kg body weight) beginning at onset of clinical signs and continued every three days until the vehicle-treated rats spontaneously recovered. Rats were weighed and scored twice daily using the following scale: 0 – no disease signs; 0.5 – distal limp tail; 1 – fully limp tail; 2 – mild paraparesis; 3 – moderate paraparesis; 3.5 – one hind leg paralyzed; 4 – both hind legs paralyzed; 5 – hind leg paralysis and incontinence; 6 – moribund, requires immediate euthanasia^28^,^38^,^64^.

Randomly selected rats with EAE were humanely euthanized at the peak of disease (day 12 post-immunization). After cardiac perfusion with saline^65^, spinal columns were removed and fixed in 10% buffered formalin. The lumbar region of the spinal cord was carefully dissected, embedded in paraffin, sectioned and stained with hematoxylin and eosin within the Pathology and Histology facility at Baylor College of Medicine. Slides were scored blindly by two investigators for the degree of immune cell infiltration into the gray matter of the spinal from three randomly selected cross-sections from each animal: 0 – none; 1 – low (1–10%); 2 – moderate (11–50%); 3 – high (51–100%). Representative photos were taken at 40× magnification with an upright Olympus BX41 microscope equipped with a QImaging QICAM Fast1394 camera and the QCapture Pro software v6.0.0.412.

### Statistical analysis

All data are expressed as means ± standard error of the mean (s.e.m). Parametric statistical methods were utilized to achieve sufficient statistical power with small sample sizes (*n* < 4); Bonferonni *post-hoc* tests were used for pairwise comparisons. All remaining analyses utilized non-parametric methods: a two-tailed Mann-Whitney test for comparisons between two groups, a Kruskal-Wallis test and Dunns *post-*hoc tests for comparisons between three or more groups; EAE data was analyzed using a Wilcoxon matched pairs test. All analyses utilized a significance level of *P* < 0.05 and were performed using Prism (GraphPad).

## Additional Information

**How to cite this article**: Huq, R. *et al*. Preferential uptake of antioxidant carbon nanoparticles by T lymphocytes for immunomodulation. *Sci. Rep.* **6**, 33808; doi: 10.1038/srep33808 (2016).

## Supplementary Material

Supplementary Information

Supplementary Movie 1

Supplementary Movie 2
