# Supplementary material for: Preferential uptake of antioxidant carbon nanoparticles by T lymphocytes for immunomodulation
Source: Sci Rep. 2016 Sep 22;6:33808. doi: 10.1038/srep33808 (PMC5031970; doi:10.1038/srep33808)
Supplement: Supplementary Information [file srep33808-s1.doc]

Title: Preferential uptake of antioxidant carbon nanoparticles by T lymphocytes for immunomodulation

Redwan Huq^1,2^, Errol L.G. Samuel^3^, William K.A. Sikkema^3^, Lizanne G. Nilewski^3^, Thomas Lee^1,4^, Mark R. Tanner^1,5^, Fatima S. Khan^1^, Paul C. Porter^6^, Rajeev B. Tajhya^1,2^, Rutvik S. Patel^1^, Taeko Inoue^1,2^, Robia G. Pautler^1^, David B. Corry^6,7^, James M. Tour^3,8^*, Christine Beeton^1,7,9^*

**SUPPLEMENTARY FIGURES**

**Supplementary Figure S1**. **Schematic illustrating protocol used for determining internalization of PEG-HCCs by immune cells *in vivo*.**

Inject rats subcutaneously with 2 mg kg^-1^ PEG-HCCs

Wash and fix cells

Stain with anti-PEG antibody

Permeabilize and stain with anti-PEG antibody

Analyze by flow cytometry

Surface-bound PEG-HCCs only

Surface-bound and intracellular PEG-HCCs

Wash cells

Stain for cell surface markers

Wait 24 hours

Isolate organs and prepare single-cell suspensions

| **Cell type** | **Spleen *ex vivo*** | **Spleen *in vivo*** | **Lymph node *in vivo*** |
| --- | --- | --- | --- |
| T cell | 8901 ± 265 | 4235 ± 25 | 5715 ± 176 |
| B cell | 4979 ± 182 | 1356 ± 34 | 4284 ± 88 |
| Neutrophil | 1764 ± 63 | 399 ± 7 | 2351 ± 13 |
| Macrophage | 1269 ± 42 | 511 ± 34 | 857 ± 19 |
| Dendritic cell | 129 ± 12 | 99 ± 1 | 304 ± 11 |
| Natural killer cell | 2007 ± 138 | 222 ± 0 | Not analyzed |

**a**

**b**

**Supplementary Figure S2**. **Flow cytometry gating strategy used for identifying rat splenic immune cell subsets.** (a) The upper left dot plot was gated on single white blood cells, which was subgated in the upper middle dot plot to show single live cells using a viability dye. Single live cells were either gated for surface markers specific for natural killer (NK) lymphocytes (CD3^–^CD161a^+^) and T cells (CD3^+^CD161a^–^), or B lymphocytes (CD3^–^B220^+^) and T cells (CD3^–^B220^+^). Non-B and -T lymphocytes (CD3^–^B220^–^) were gated for a surface marker used to identify neutrophils (CD3^–^B220^–^Ly6G^+^). Non-neutrophils (CD3^–^B220^–^Ly6G^–^) were further subgated for surface markers of macrophages (CD3^–^B220^–^Ly6G^–^CD11b^+^CD103^–^) and dendritic cells (CD3^–^B220^–^Ly6G^–^CD11b^+^CD103^+^). (b) Number of cells in each of the populations gated in a.

**a spleen from vehicle-treated rat**


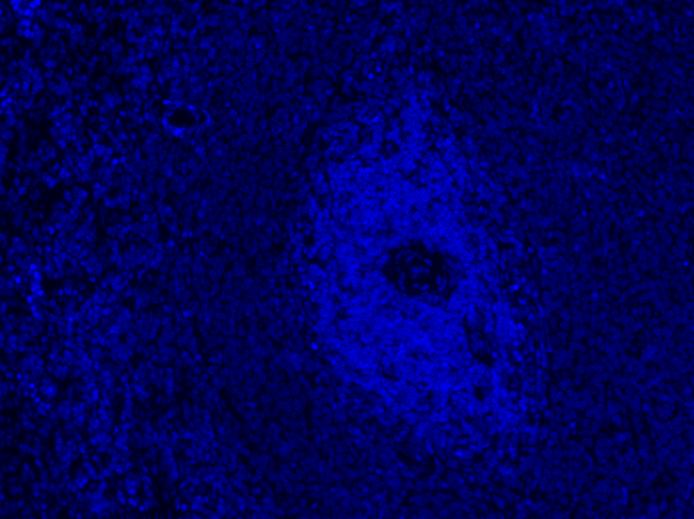

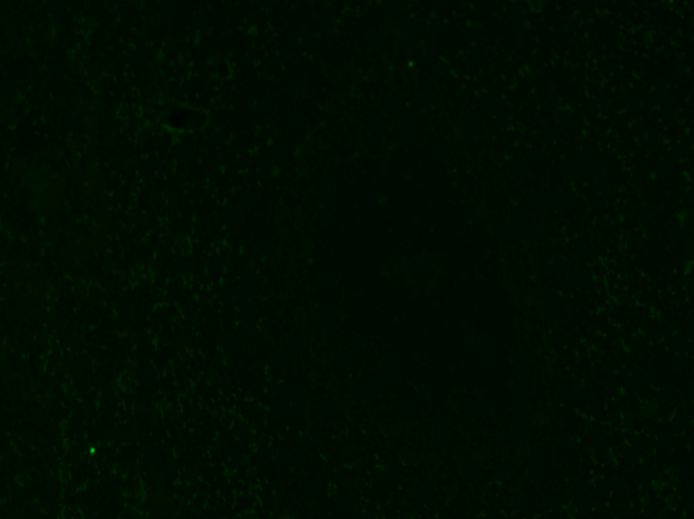


Cell nuclei

PEG


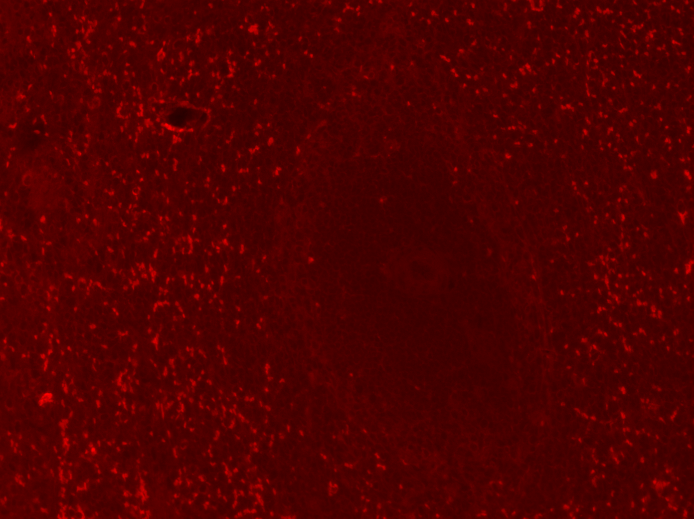

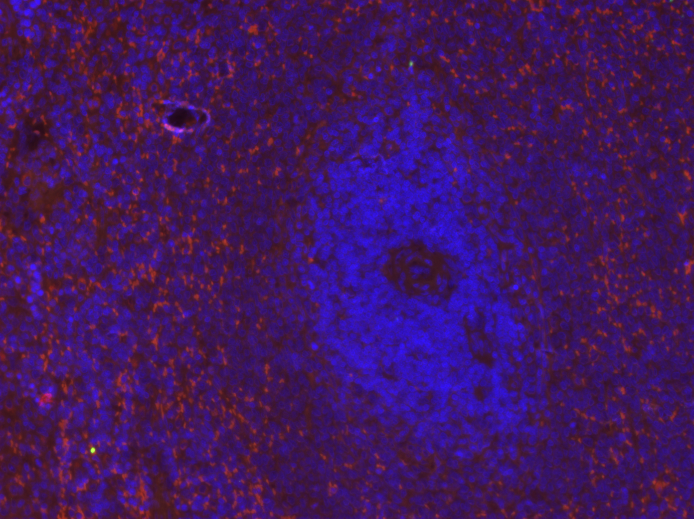


CD3 (T cells)

Overlay

Follicle (B cell zone)

T cell zone

**b spleen from PEG-HCC-treated rat**


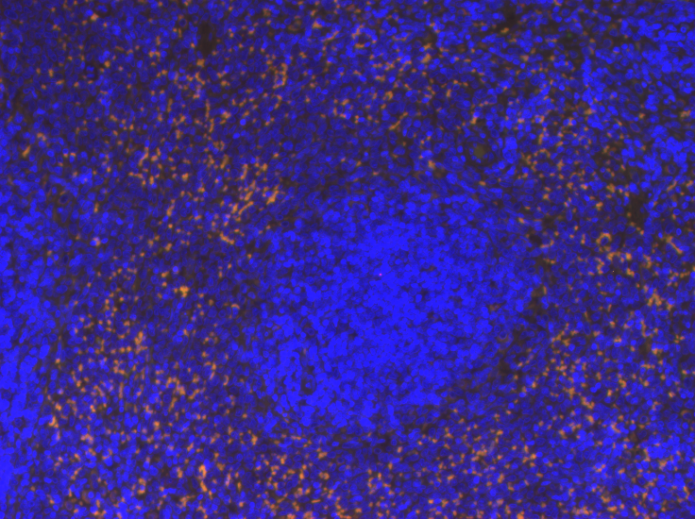

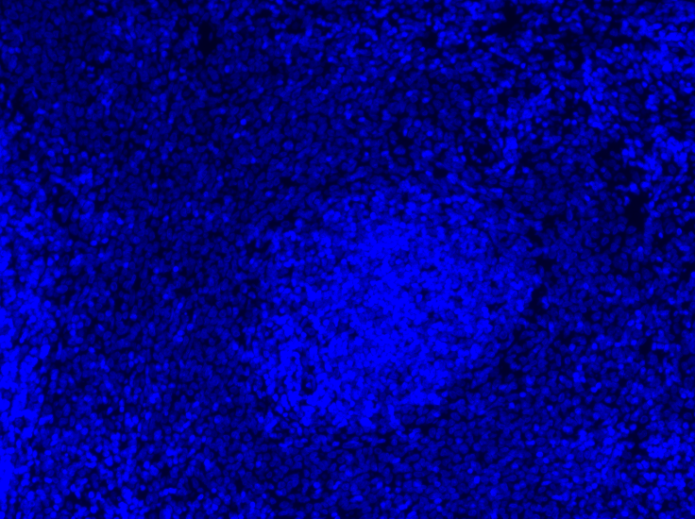

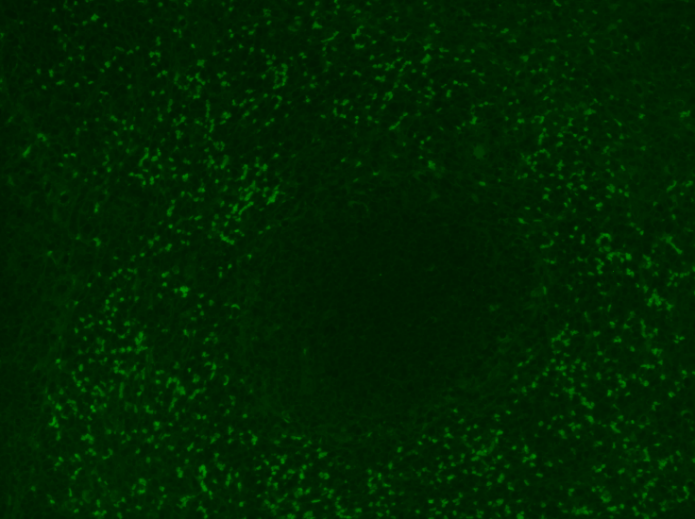

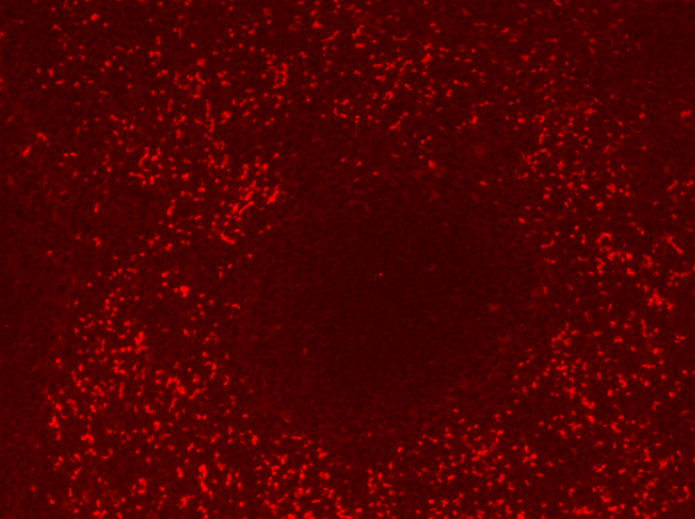


Cell nuclei

PEG

CD3 (T cells)

Overlay

Follicle (B cell zone)

T cell zone

**Supplementary Figure S3. PEG-HCCs co-localize with T cells in the rat spleen.** PEG-HCC localization, analyzed by immunohistochemistry, in white pulp regions of spleen collected from (a) rats injected with PBS or (b) 2 mg/kg body weight PEG-HCCs 24 h prior (*n* = 3 rats). PEG-HCCs were distinctly found in the T cell-rich periarteriolar lymphoid sheaths, surrounding B cell-rich follicles that were devoid of the nanoparticles, in spleens of PEG-HCC-injected animals. Cell nuclei (blue) identified with DAPI, PEG-HCCs (green) identified with anti-PEG Ab and Alexa Fluor 488-labeled conjugate antibody, T cells (red) identified with anti-CD3 antibody and Alexa 568-labeled conjugate antibody. Scale bars, 100 μm.


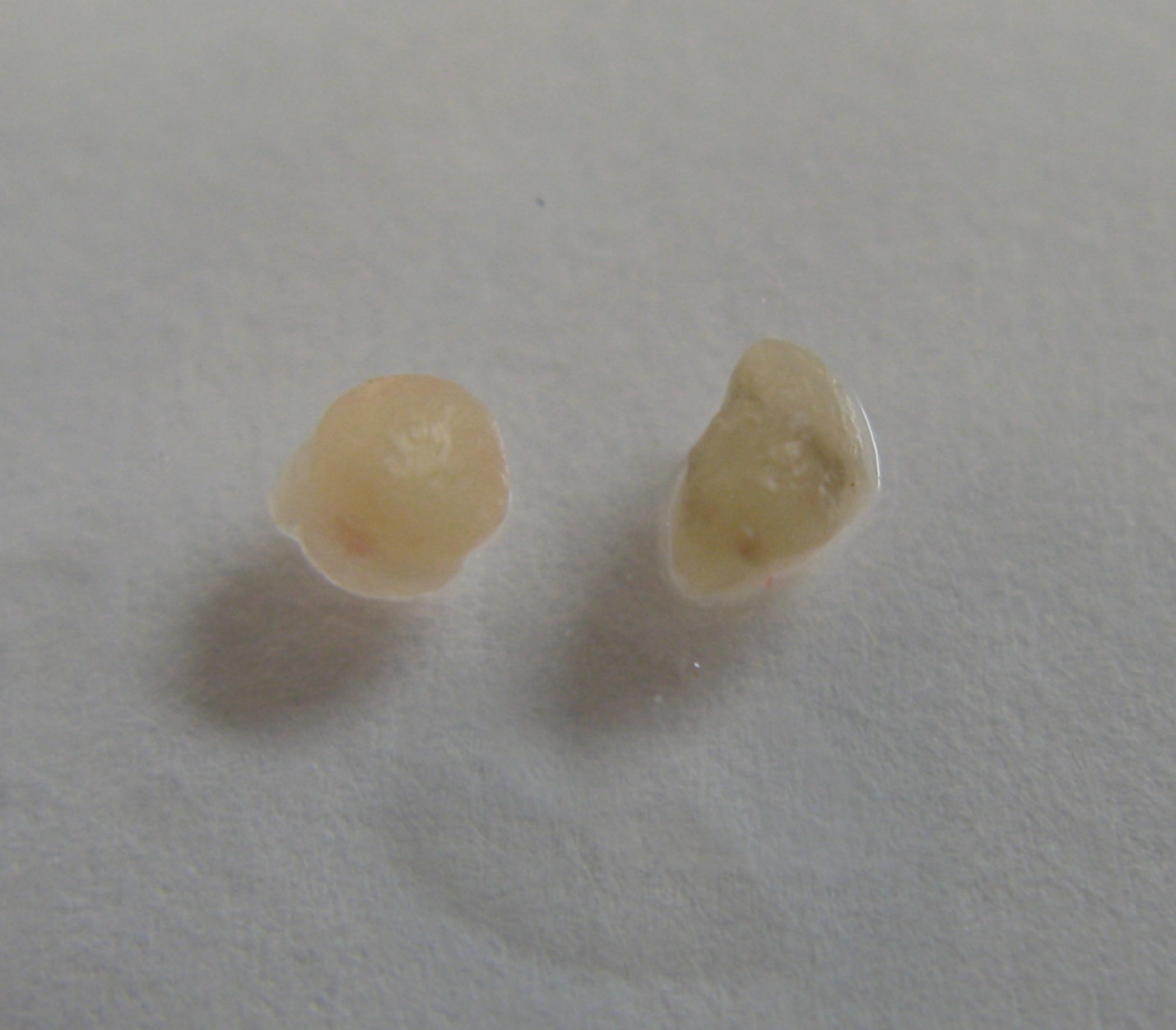
 **Supplementary Figure S4. PEG-HCCs are present in the lymph nodes after injection into rats.** Photographs of inguinal lymph nodes isolated from rats 24 h after injection with PBS (left) or 2 mg/kg body weight PEG-HCCs (right). PEG-HCCs are visually observable in the lymphatics as dark stripes.

PBS

PEG-HCCs

**Supplementary Figure S5. PEG moieties do not detach from HCCs in physiological conditions.** Percentage of nanoparticles with PEG moieties as determined by thermogravimetric analysis after incubation with water or freshly collected rat serum for 0, 1 or 24 h (*n* = 3 experiments). The lack of PEG decomposition in physiological conditions suggests that the PEG moieties remain attached to the nanoparticles and therefore will be detectable using an anti-PEG antibody.

PEG

Relative T cell count

Anti-PEG Ab

Secondary Ab

No PEG-HCCs

No anti-PEG Ab

Secondary Ab

PEG-HCCs

Anti-PEG Ab

Secondary Ab

PEG-HCCs

PEG-HCC^+^

PEG-HCC^+^

PEG-HCC^+^

**a**

**b**

**Supplementary Figure S6. FCM-based technique to measure PEG-HCC uptake does not elicit a positive signal in the absence of PEG-HCCs or anti-PEG antibody.** (a) Representative FCM histograms graphically showing that no PEG-HCCs were detected in intact (gray) or permeabilized (black line) rat splenic T cells in the absence of PEG-HCCs while using both a primary anti-PEG antibody and secondary conjugate antibody (left). A similar result was obtained in the presence of PEG-HCCs and the secondary antibody, and in the absence of the ant-PEG antibody (middle). However, when PEG-HCCs (0.1 μg/mL) and both antibodies are present, the nanoparticles were indeed detected (right); permeabilization provided a stronger positive signal indicative of nanoparticle internalization. (b) Quantification of FCM data (*n* = 3 spleen preparations) in rat splenic T cells.

**Supplementary Figure S7. PEG-HCCs are not detectable in dead T cells.** Quantification of FCM data of rat splenic T cells treated with 0.1 μg/mL PEG-HCCs and stained with a viability dye (*n* = 3 splenic preparations). Cells were gated on single live or dead T cells as shown in Supplementary Fig. S2.

**a**

**b**

**Supplementary Figure S8. Representative contour plots for kinetics of PEG-HCCs internalization and loss from T cells.** (a) Representative FCM contour plots of rat splenocytes showing the detection of PEG-HCCs associated with permeabilized T cells, using expression levels of CD3 (x-axis) and of PEG (y-axis), incubated with the nanoparticles for the indicated time points (*n* = 3 splenic preparations; 16000 live T cell singlets analyzed per sample). Intracellular PEG-HCC levels in T cells saturated approximately after a 25 min incubation. (b) FCM contour plots of rat splenocytes showing PEG-HCCs associated with permeabilized T cells at the indicated time points after a 30 min incubation and washing of the cells to remove excess nanoparticles. After 6 h post-incubation and wash, PEG-HCCs were nearly undetectable in T cells (*n* = 3 splenic preparations; 18000 live T cell singlets analyzed per sample).

**
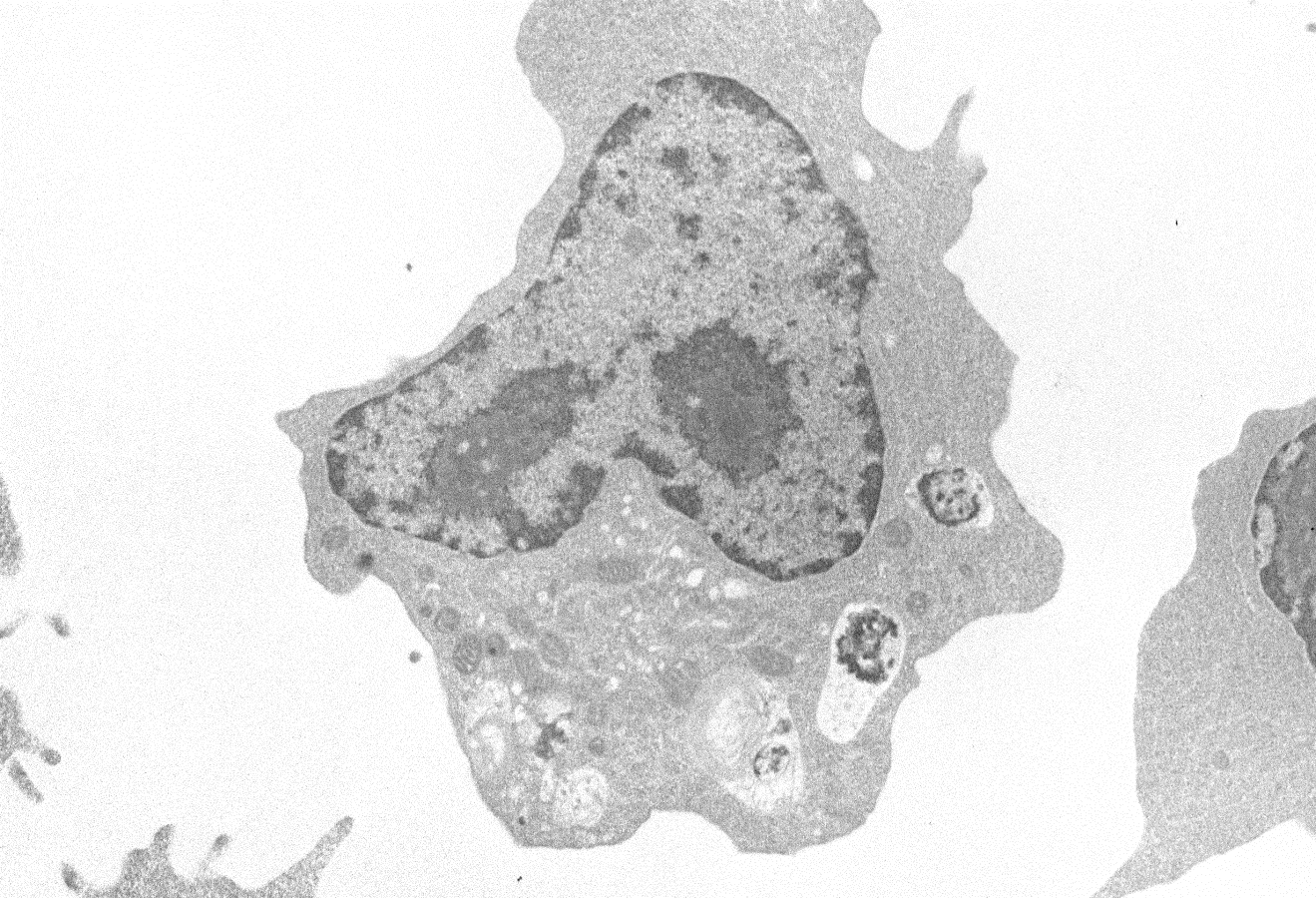
**

2 μm

**a**

**Nu**

**AV**

400 nm

**M**

**M**

**E**

**CM**

**LB**

**INSET**

**
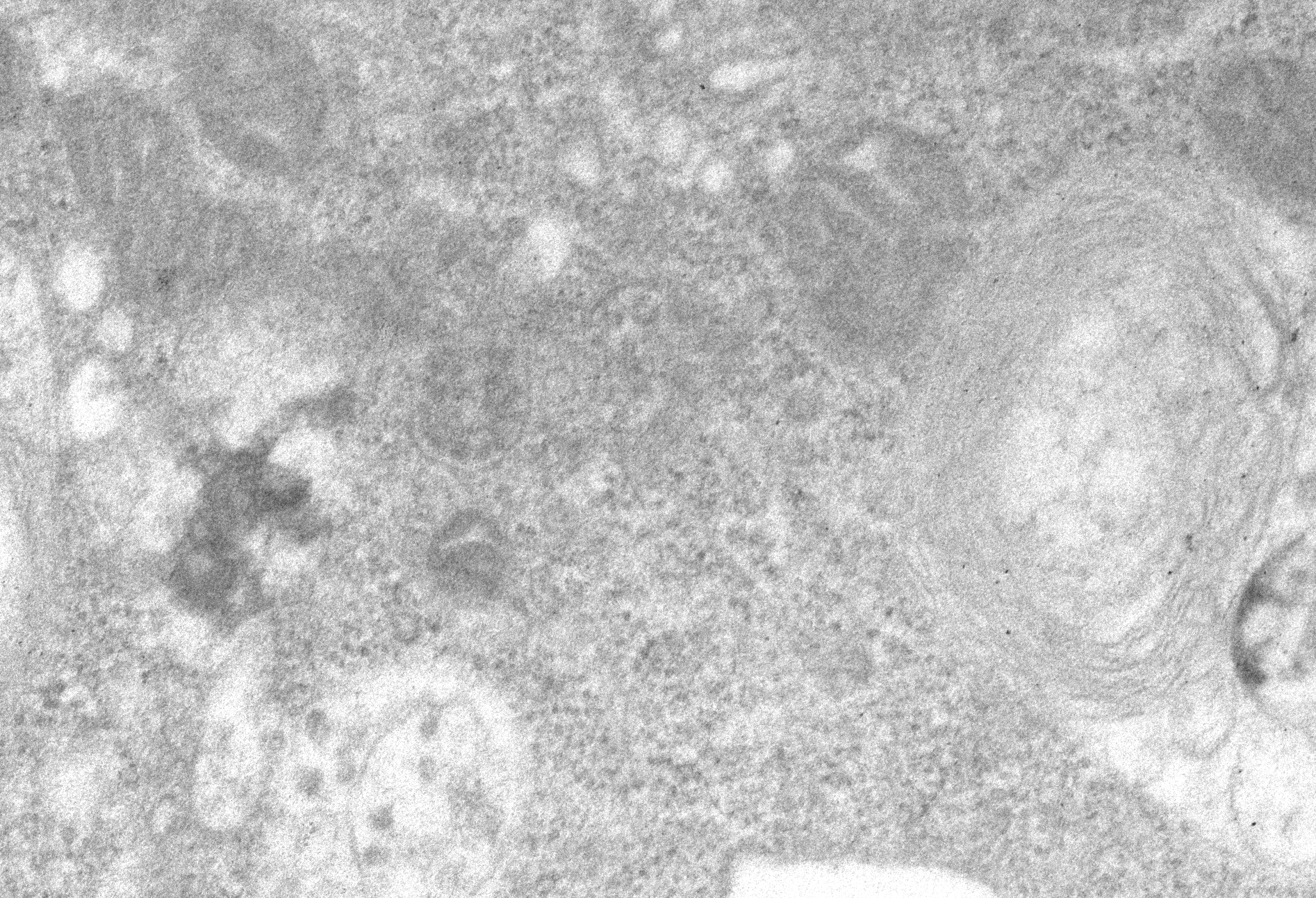
**

**
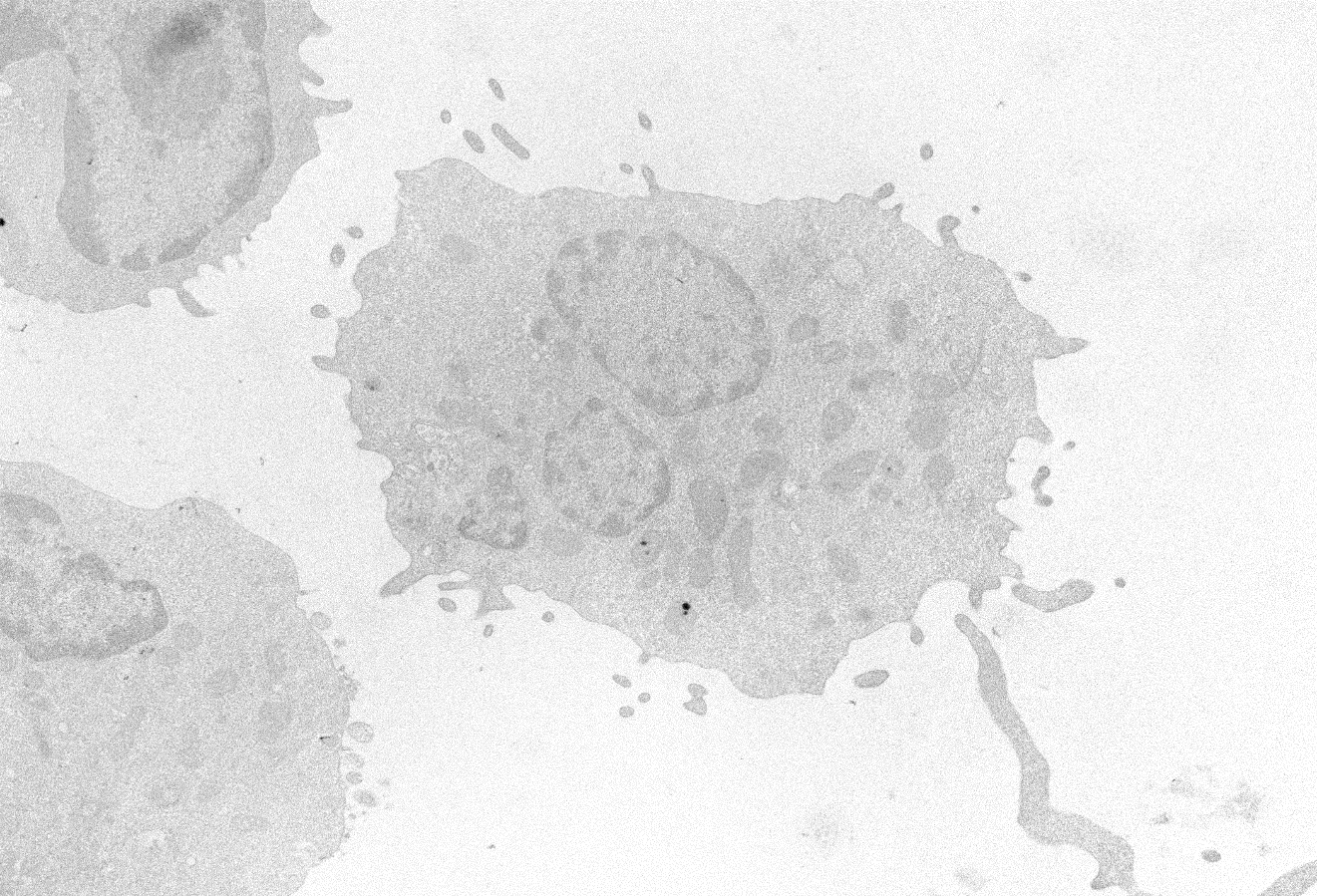

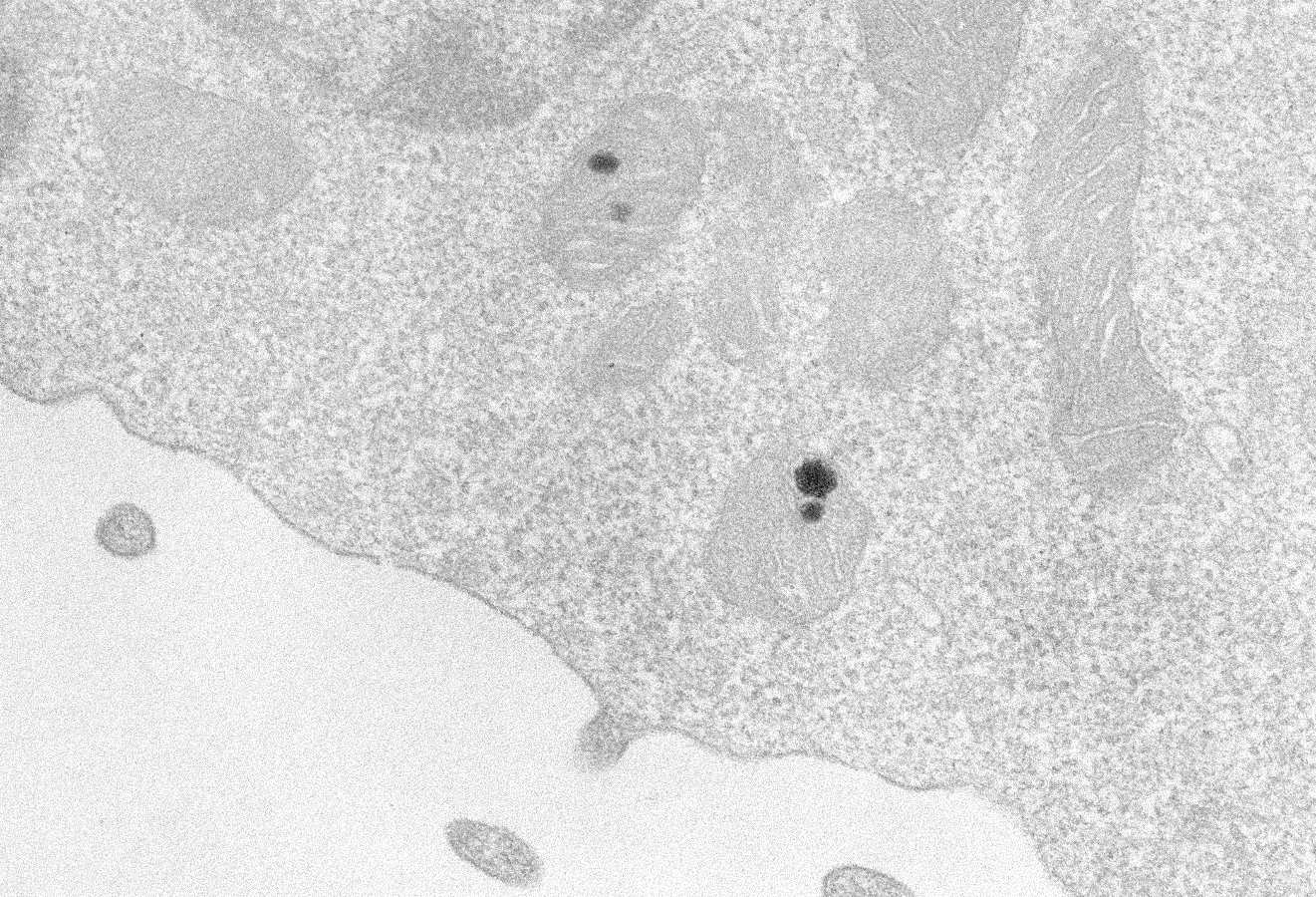
**

2 μm

**b**

400 nm

**Nu**

**M**

**M**

**CM**

**INSET**


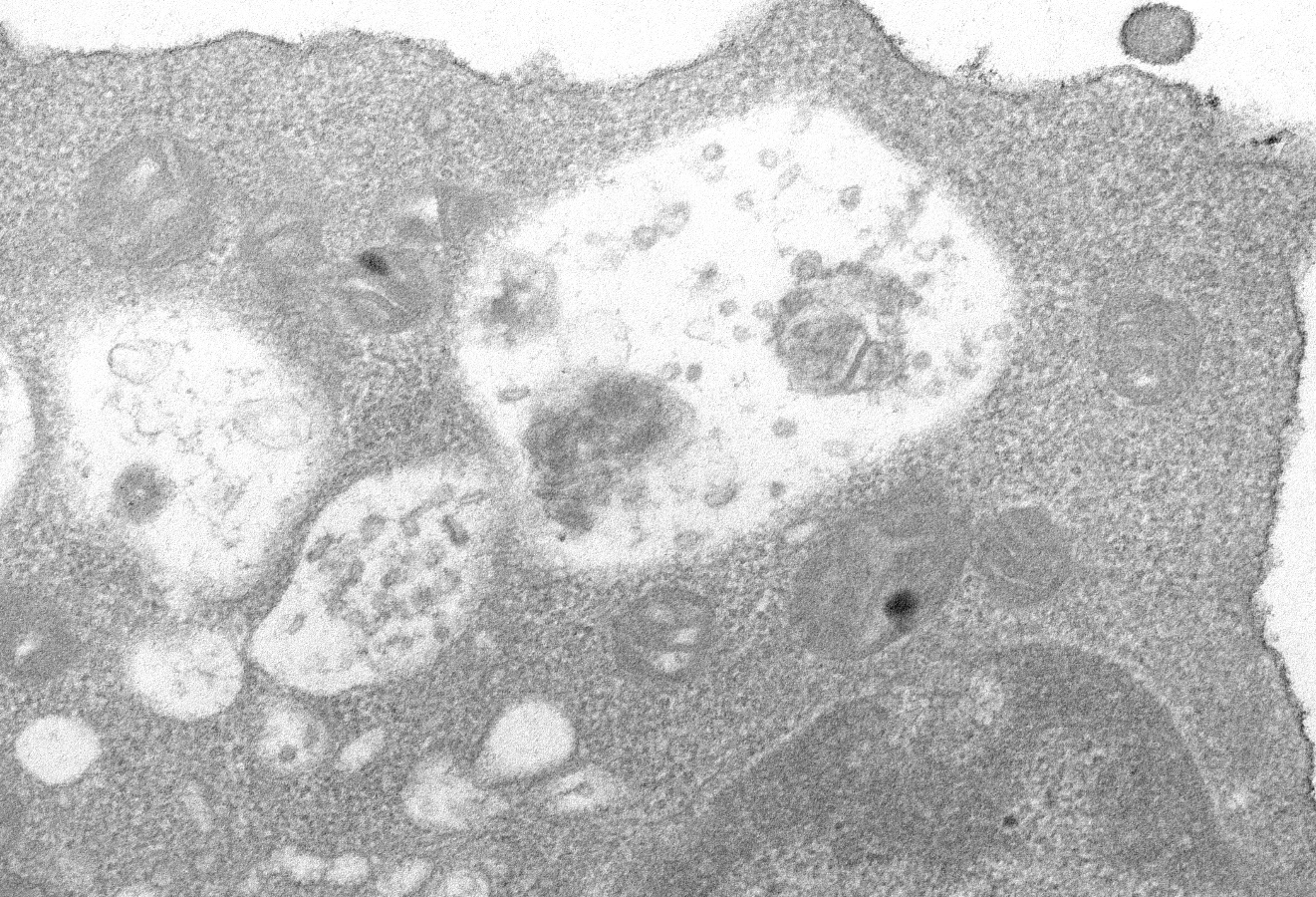

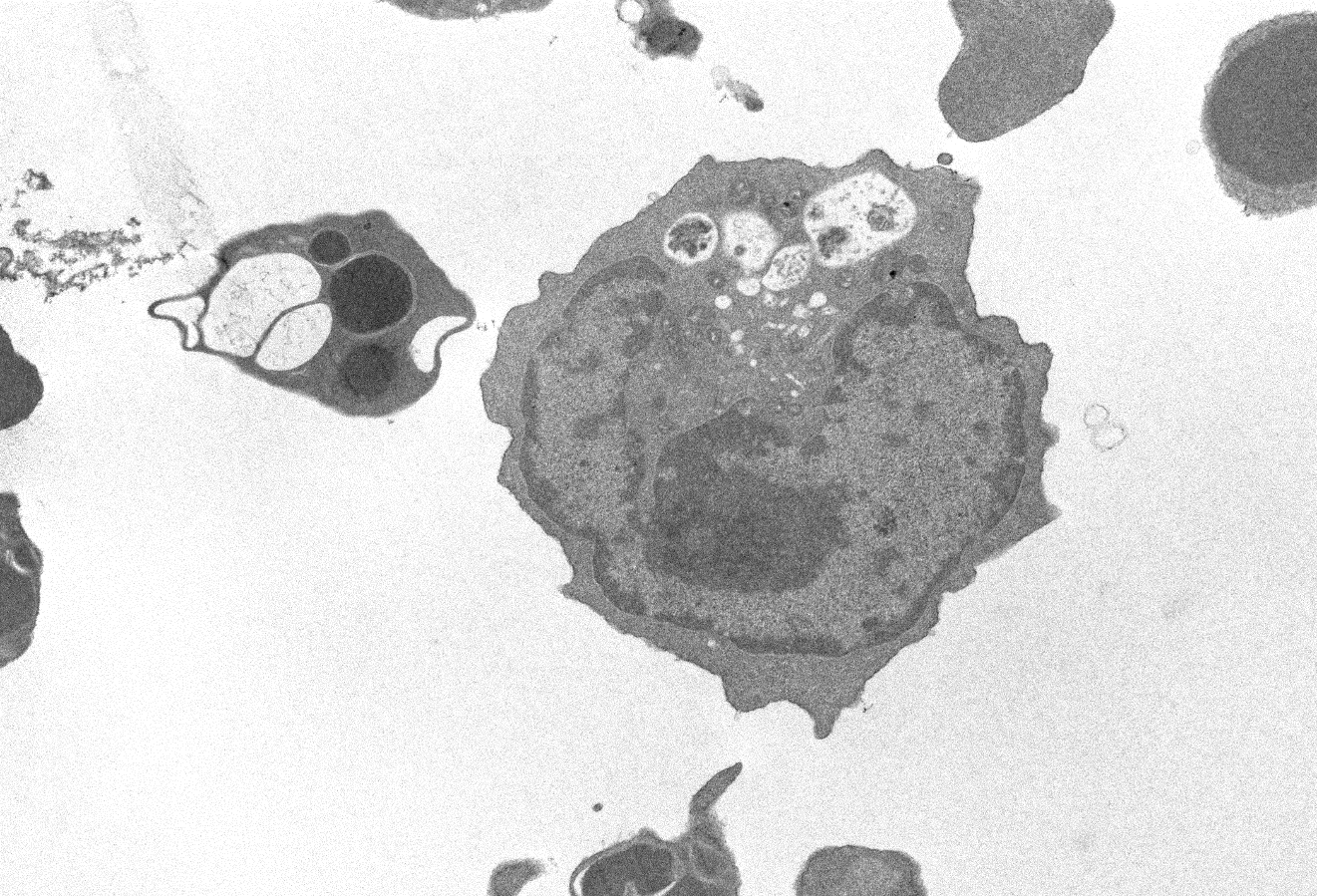


2 μm

**c**

**Nu**

400 nm

**E**

**E**

**Nu**

**M**

**M**

**CM**

**INSET**

**Supplementary Figure S9. High resolution micrographs of subcellular localization of PEG-HCCs inside T cells.** Subcellular localization of PEG-HCCs in T cells, visualized by transmission electron microscopy, that were either (a) untreated or (b) incubated with a low (0.1 μg/mL) or (c) high (10 μg/mL dose of PEG-HCCs. Arrows point to bundles of PEG-HCCs in the mitochondria and at high doses, also associating with the cell membrane. Nucleus (Nu); mitochondrion (M); cell membrane (CM); lamellar body (LB); endosome (E); autophagic vesicle (AV).

**Supplementary Figure S10. Schematic illustrating cell-based sandwich ELISA protocol used for detecting exit/degradation of PEG-HCCs from T cells.**

Coat plate with anti-PEG antibody

Incubate plate for 5 h

Discard cells and wash plate

Add biotinylated anti-PEG antibody

Incubate overnight

Add PEG-HCC-treated cells to plate

Wash cells

Incubate T cells with PEG-HCCs

Wash plate and add streptavidin-AKP

Wash plate and add PNPP substrate

Add NaOH to halt reaction

Read absorbance at 405 nm

**a**

**b**

**c**

HCCs

PEG-HCCs

**
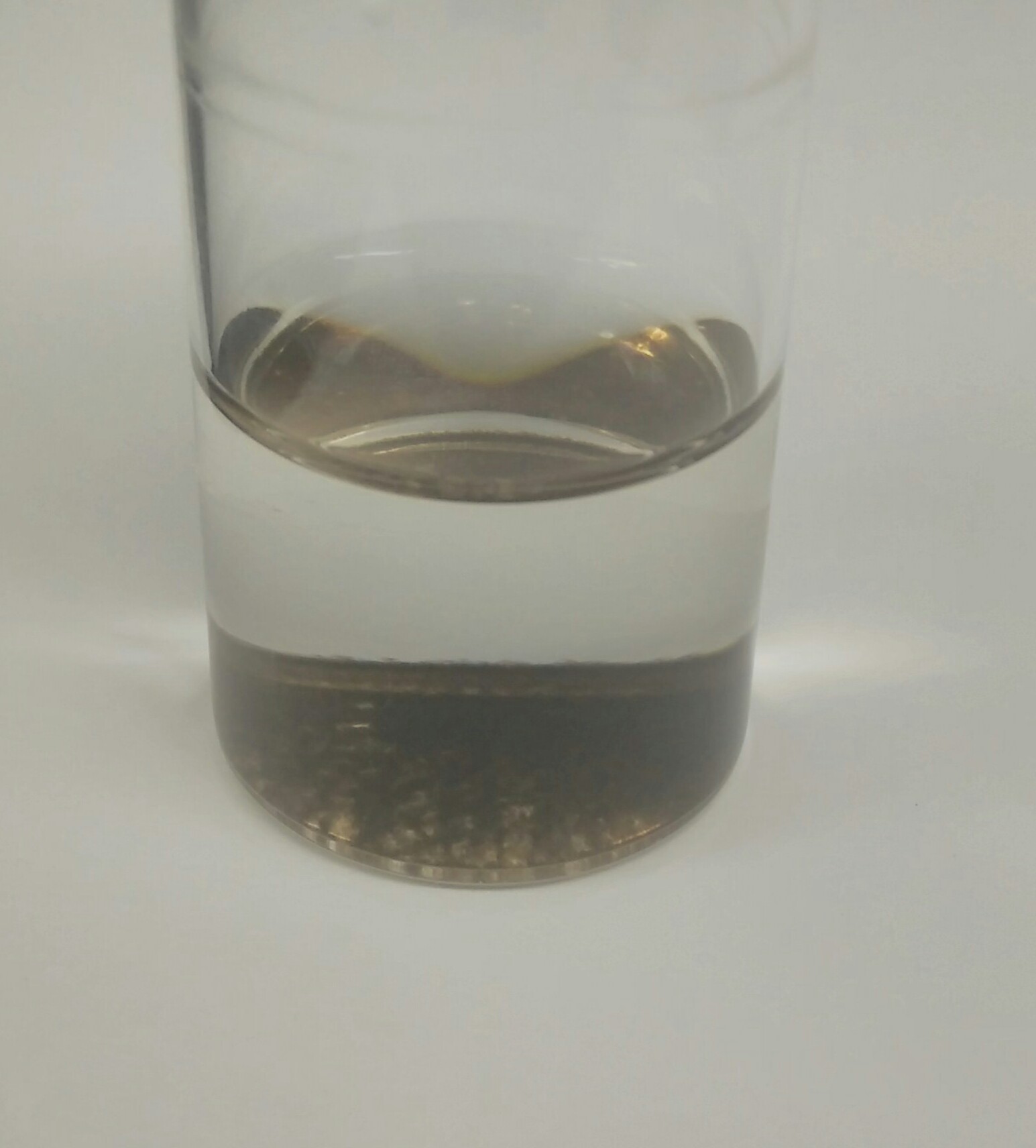

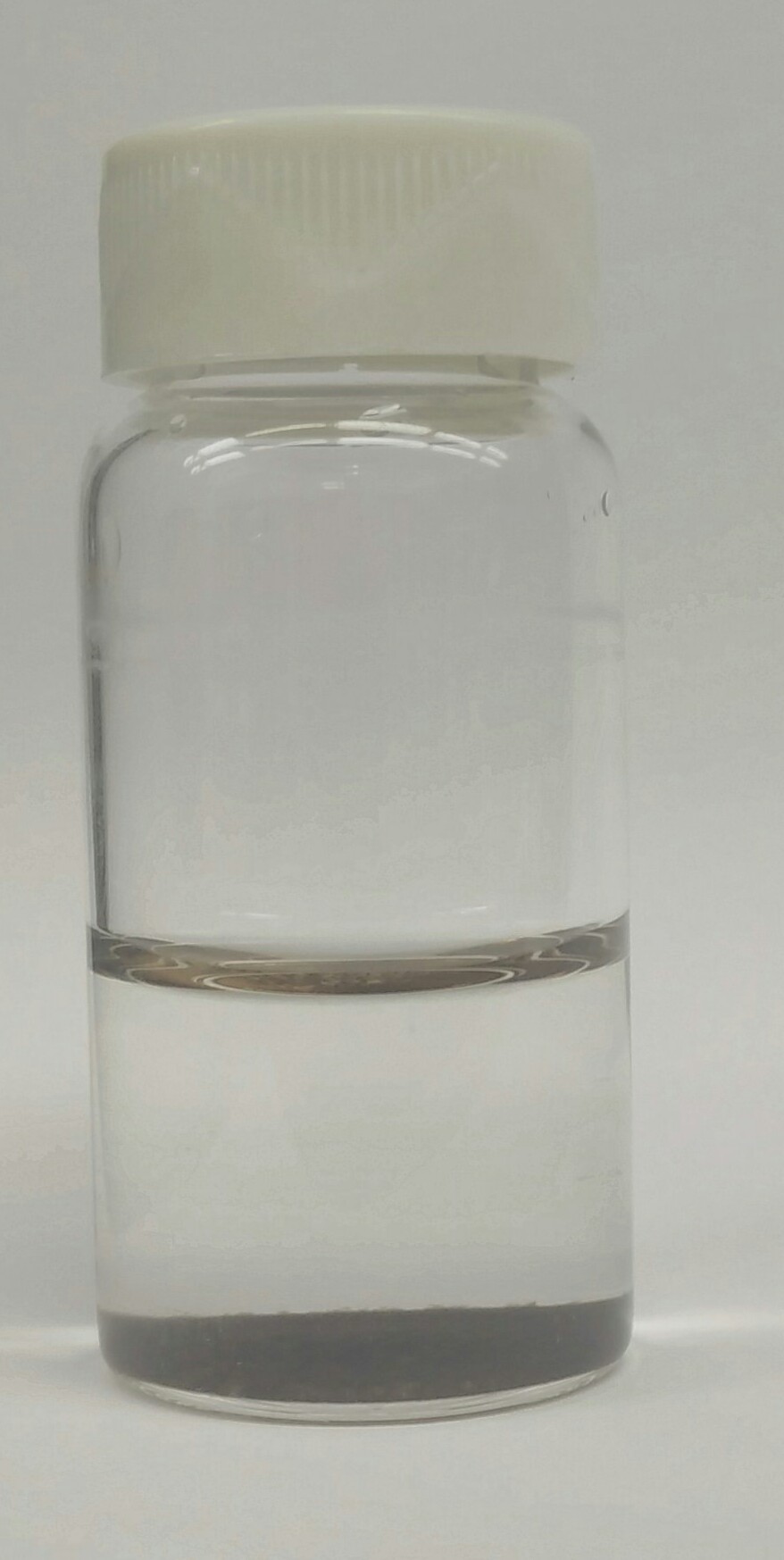

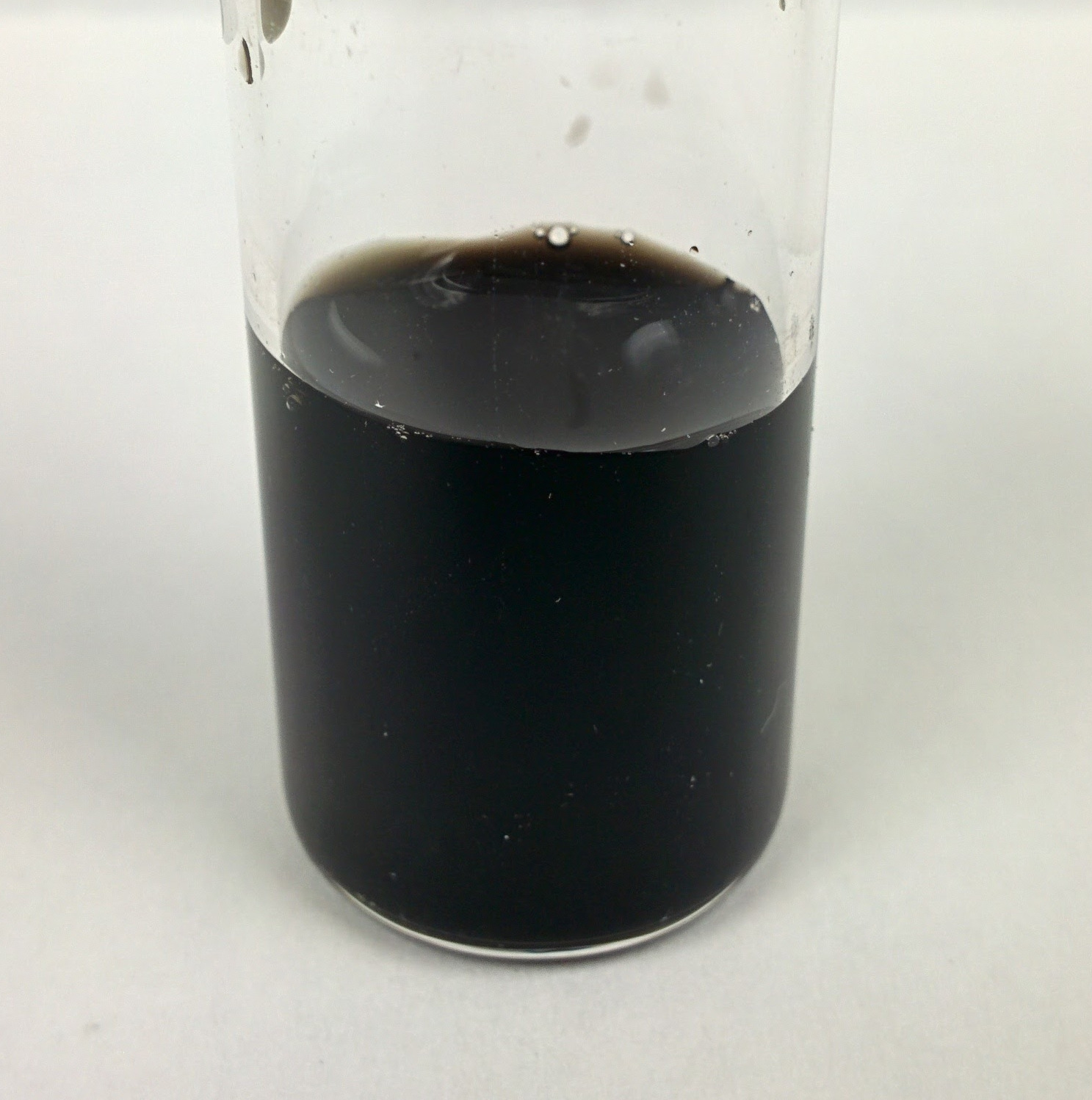

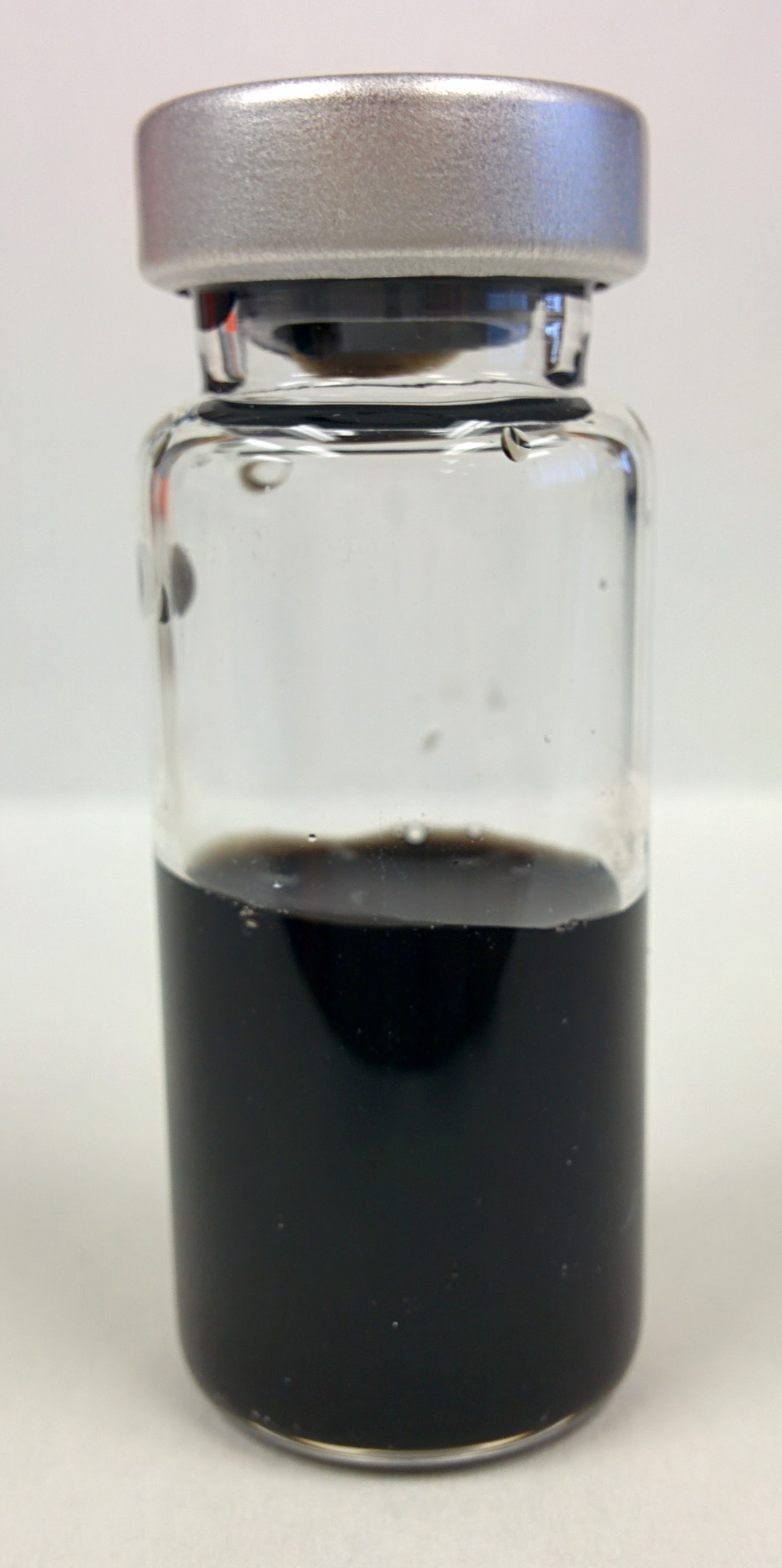
**

**Supplementary Figure S11. HCCs and PEG-HCCs inhibit proliferation of myelin basic protein-specific T cells without inducing T cell death.** (a) Proliferation of antigen-stimulated myelin basic protein-specific T cells, measured by [^3^H] thymidine incorporation, in the presence of the indicated concentrations of HCCs or PEG-HCCs. HCCs reduced T cell proliferation in a similar fashion as PEG-HCCs. Unstimulated cells are shown as controls (*n* = 3 replicates). (b) Quantification of cell death by FCM in myelin basic protein-specific T cells left unstimulated, antigen-stimulated, incubated with PEG-HCCs or HCCs prior to antigen-stimulation, or antigen-stimulated and treated with staurosporine (*n* = 3 replicates). Neither PEG-HCCs nor HCCs have cytotoxic effects on myelin basic protein-specific T cells. (c) Photographs of HCCs (left) or PEG-HCCs (right) in PBS after 1 day without disturbance. HCCs flocculate and fall to bottom of vial, while PEG-HCCS remain in solution. Data are expressed as means ± s.e.m. **P* < 0.05, ***P* < 0.01.

**a**

**b**

**Supplementary Figure S12. PEG-HCCs inhibit proliferation of T cells from human peripheral blood or rat spleen after mitogen stimulation.** (a) Proliferation of human peripheral blood T cells stimulated by the T cell mitogen phytohemagglutinin, measured by [^3^H] thymidine incorporation, in the presence of the indicated concentrations of PEG-HCCs. Unstimulated cells are shown as controls (*n* = 3 replicates). (b) Proliferation of rat splenic T cells stimulated by the T cell mitogen concanavalin A, measured by [^3^H] thymidine incorporation, in the presence of the indicated concentrations of PEG-HCCs. Unstimulated cells are shown as controls (*n* = 4 experiments). Data are expressed as means ± s.e.m. **P* < 0.05, ***P* < 0.01, ****P* < 0.001.

**Supplementary Figure S13**. **mPEG-NH_2_ alone is not sufficient to decrease the proliferation of T lymphocytes.** Proliferation of antigen-stimulated ovalbumin-specific rat T cells, measured by [^3^H] thymidine incorporation, in the presence of the indicated concentrations of mPEG-NH_2_ (5000 kDa). Unstimulated cells and antigen-stimulated/PEG-HCC-treated cells are shown as controls (*n* = 6 replicates).

IL-17A

SSC

Unstimulated

Stimulated

100 μg/mL PEG-HCCs

+ Stimulated

**Supplementary Figure S14.** **FCM contour plots showing the detection of proinflammatory cytokines IL-2, IFN-γ and IL-17A in T cells treated with PEG-HCCs.** Intracellular detection by FCM of the Th1 cytokines IL-2 (top) and IFN-γ (middle) in antigen-stimulated GFP-transduced T cells incubated with PEG-HCCs. Intracellular detection of the Th17 cytokine IL-17A (bottom) in lymph node T cells collected from rats with acute experimental autoimmune encephalomyelitis, incubated with PEG-HCCs and stimulated with PMA and ionomycin. Unstimulated cells are shown as controls.

**Supplementary Figure S15**. **PEG-HCCs do not affect homeostatic proliferation of unstimulated T lymphocytes.** Proliferation of unstimulated ovalbumin-specific rat T cells, measured by [^3^H] thymidine incorporation, in the presence of the indicated concentrations of PEG-HCCs. Unstimulated/untreated cells and antigen-stimulated/untreated cells are shown as controls (*n* = 6 replicates).

**Supplementary Figure S16. Phagocytic index of rat macrophages treated with PEG-HCCs.** Histogram of splenic rat macrophages phagocytizing various number of zymosan bioparticles after incubation with the indicated concentrations of PEG-HCCs.

Load rat macrophages with ovalbumin

Wash cells, remove excess antigen

Measure T cell proliferation

Add ovalbumin-specific rat T cells to macrophages

PEG-HCCs

PEG-HCCs

**Supplementary Figure S17**. **Methodology used to identify whether PEG-HCCs affect the antigen processing and presentation by macrophages.** Proliferation of T cells in the presence of macrophages, serving as antigen-presenting cells and incubated with PEG-HCCs either upon antigen loading (green) or upon addition of T cells to macrophages (blue), used as a readout for adequate antigen processing and presentation.

**Supplementary Figure S18. PEG-HCCs alone do not induce fungistasis of *Aspergillus* *niger*.** Enumeration of hyphal growth after *Aspergillus niger* was treated with the indicated doses of PEG-HCCs for 24 h at 37°C and 5% CO_2_ (*n* = 3 replicates).

**SUPPLEMENTARY TABLES**

| Endotoxin concentration | 0.184 EU/mL at PEG-HCC concentration 100 μg/mL |
| --- | --- |
| PEG-HCC size | 3 nm × 35 nm |
| PEG-HCC hydrodynamic size | 50 nm |
| Target ROS | O_2_^•–^, HO^•^ |
| Number of ROS removed per PEG-HCC | Estimated 10^6^ |
| ROS generated | H_2_O_2_ |
| Required detoxifying enzyme | None |

**Table S1.** Endotoxin contribution from PEG-HCCs injected into animals are below the acceptable limits for animals^1^. PEG-HCC characterization is summarized below from previous findings^2,3^.

Primary antibodies used for flow cytometry and immunohistochemistry.

| **Markers** | **Conjugation** | **Vendor** | **Catalog number** |
| --- | --- | --- | --- |
| CD3 | APC | BD Pharmingen | 557030 |
| CD3 | Brilliant Violet 605 | BD Pharmingen | 563949 |
| CD3 | n/a | eBioscience | 14-0030-85 |
| B220 | PE | eBioscience | 12-0460-82 |
| PEG | n/a | Abcam | Ab51257 |
| PEG | biotin | Abcam | Ab53449 |
| CD11b | V450 | BD Pharmingen | 562108 |
| CD103 | Alexa Fluor 647 | Biolegend | 205509 |
| CD161a | PE | BD Pharmingen | 555009 |
| Ly6G | FITC | Abcam | ab25024 |
| IL-2 | n/a | Life Technologies | ARC0022 |
| IFN-γ | biotin | Biolegend | 510901 |
| IL-17A | eFluor 450 | eBioscience | 48-7177-82 |
| CD4 | V450 | BD Pharmingen | 561579 |
| CD8 | PE | BD Pharmingen | 554857 |
| CD62L | APC | Biolegend | 202916 |
| γδ TCR | PE | BD Pharmingen | 551802 |

Secondary antibodies used for flow cytometry and immunohistochemistry.

| **Target** | **Conjugate** | **Vendor** | **Catalog number** |
| --- | --- | --- | --- |
| streptavidin | PE | BD Pharmingen | 554061 |
| streptavidin | AKP | BD Pharmingen | 554065 |
| rabbit IgG | Alexa Fluor 488 | Life Technologies | A11034 |
| rabbit IgG | Alexa Fluor 647 | Life Technologies | A21245 |
| rabbit IgG | Alexa Fluor 750 | Life Technologies | A21039 |
| rabbit IgG | Pacific Blue | Life Technologies | P10994 |
| mouse IgG | Alexa Fluor 568 | Life Technologies | A10037 |

**Table S2**. List of antibodies and their fluorophore conjugate, if applicable, used for FCM and immunohistochemical analysis.

**SUPPLEMENTARY MOVIES**

**Movie S1**. Severe paraparesis is observed in rats with acute active EAE treated with PBS (Vehicle) starting at onset of clinical signs and continued every 3 days for the duration of the disease.

**Movie S2**. Mild to no paraparesis is observed in rats with acute active EAE treated with 2 mg/kg body weight PEG-HCCs starting at onset of clinical signs and continued every 3 days for the duration of the disease.

**SUPPLEMENTARY REFERENCES**

1. Malyala, P. & Singh, M. Endotoxin limits in formulations for preclinical research. *J. Pharm. Sci.* **97**, 2041-2044 (2007).

2. Berlin, J. M.*, et al.* Effective drug delivery, in vitro and in vivo, by carbon-based nanovectors noncovalently loaded with unmodified Paclitaxel. *ACS nano* **4**, 4621-4636 (2010).

3. Samuel, E. L.*, et al.* Hydrophilic carbon clusters as therapeutic, high-capacity antioxidants. *Trends Biotechnol.* **32**, 501-505 (2014).
